# Avocado-derived polyols for use as novel co-surfactants in low energy self-emulsifying microemulsions

**DOI:** 10.1038/s41598-020-62334-y

**Published:** 2020-03-27

**Authors:** Nawaz Ahmed, Behnoush Kermanshahi, Saeed M. Ghazani, Katrina Tait, Matthew Tcheng, Alessia Roma, Shannon P. Callender, Richard W. Smith, William Tam, Shawn D. Wettig, Michael A. Rogers, Alejandro G. Marangoni, Paul A. Spagnuolo

**Affiliations:** 1 Department of Food Science, University of Guelph, Guelph, Ontario N1G 2WI Canada; 2 School of Pharmacy, University of Waterloo, Waterloo, Ontario N2L 3G1 Canada; 3 University of Waterloo Mass Spectrometry Facility, Department of Chemistry, 200 University Avenue West, Waterloo, ON N2L 3G1 Canada; 4 Guelph-Waterloo Centre for Graduate Work in Chemistry and Biochemistry, Department of Chemistry, University of Guelph, Guelph, Ontario N1G 2W1 Canada; 5 Waterloo Institute for Nanotechnology, University of Waterloo, 200 University Ave. W., Waterloo, Ontario N2L 3G1 Canada

**Keywords:** Drug development, Drug delivery

## Abstract

Avocado (*Persea americana* Mill.; Lauraceae) seed-derived polyhydroxylated fatty alcohols (PFAs) or polyols (i.e., avocadene and avocadyne) are metabolic modulators that selectively induce apoptosis of leukemia stem cells and reverse pathologies associated with diet-induced obesity. Delivery systems containing avocado polyols have not been described. Herein, natural surface active properties of these polyols are characterized and incorporated into self-emulsifying drug delivery systems (SEDDS) that rely on molecular self-assembly to form fine, transparent, oil-in-water (O/W) microemulsions as small as 20 nanometers in diameter. Mechanistically, a 1:1 molar ratio of avocadene and avocadyne (i.e., avocatin B or AVO was shown to be a eutectic mixture which can be employed as a novel, bioactive, co-surfactant that significantly reduces droplet size of medium-chain triglyceride O/W emulsions stabilized with polysorbate 80. *In vitro* cytotoxicity of avocado polyol-SEDDS in acute myeloid leukemia cell lines indicated significant increases in potency and bioactivity compared to conventional cell culture delivery systems. A pilot pharmacokinetic evaluation of AVO SEDDS in C57BL/6J mice revealed appreciable accumulation in whole blood and biodistribution in key target tissues. Lastly, incorporation of AVO in SEDDS significantly improved encapsulation of the poorly water-soluble drugs naproxen and curcumin.

## Introduction

Avocados have been shown to be an exclusive source of unique long carbon chain (C_17_ to C_21_) polyhydroxylated fatty alcohols or polyols that possess anti-cancer, anti-inflammatory and anti-microbial properties^1–4^. Previously, we demonstrated that avocatin B (AVO), a 1:1 mixture of two 17-carbon polyols (avocadyne and avocadene), target mitochondria and inhibit fatty acid oxidation (FAO) which selectively induces death in leukemia cells^5–8^. More recently, avocadyne has been shown to exert potent immunomodulatory effects that impede dengue virus replication *in vitro*^9^. Furthermore, our laboratory determined that AVO inhibited FAO in skeletal muscle and pancreatic β-cells reversing insulin resistance and restoring glucose tolerance in diet-induced obesity^10^.

The highly promising indications for avocado polyols in human disease and nutrition necessitate the complete characterization of their physicochemical properties and subsequent development of optimal formulations for future *in vivo* and clinical studies. To our knowledge, use of avocado polyols is prominent in topical cosmetic formulations described briefly in patent literature^11,12^. This is the first report that avocadene and avocadyne possess unique physicochemical properties ideal for SEDDS applications.

SEDDS comprise a mixture of oils, co-solvents, surfactants and co-surfactants that spontaneously self-assemble into oil-in-water (O/W) or water-in-oil (W/O) nano (~100–400 nm in diameter) or micro (<100 nm in diameter) emulsions^13^. Surfactants delay droplet coalescence by reducing the surface free energy associated with the oil-water interface by creating a rigid, viscous mono-layer at the interface. Self-emulsifying microemulsions require ultra-low interfacial tension that is achievable to some extent with the use of co-surfactants (e.g., polyols like glycerol and propylene glycol or short chain alcohols like ethanol)^14^. Generally, use of polyols and short chain alcohols in microemulsions “tunes” two critical interfacial parameters. First, the amphiphilic nature of polyols leads to their spontaneous self-assembly at the oil-water interface where the polar portion of the polyol aligns towards the aqueous phase and the apolar aliphatic chain aligns towards the oil phase, which collectively modifies film curvature. Second, the self-assembly of polyols at the interface increases the elasticity of the interfacial film (referred to as the bending moduli)^15–17^.

Microemulsions as delivery-vehicles for cosmetic, food and pharmaceutical formulations are of great interest due to their low energy requirements (spontaneous formation), thermodynamic stability, low viscosity and high solubilization capacity^18,19^. However, translation of microemulsions into practice is limited due to high surfactant concentrations typically required, the need for co-surfactants (exceeding 10%), and low drug loading efficiency. In this study, we tested the ability of avocadene, avocadyne and their mixtures to spontaneously form O/W nano and microemulsions when incorporated with polysorbates and medium chain triglyceride oils. Using differential scanning calorimetry (DSC) and powder X-ray diffraction (XRD) we explored the unique physical properties of avocado polyols that enable their molecular self-assembly in SEDDS. Further, we examined if avocado polyol SEDDS have enhanced bioactivity *in vitro* and if they accumulate in blood and key target tissues when delivered orally to mice. Lastly, we tested the ability of AVO to act as a novel co-surfactant and its impact on droplet size of poorly water-soluble drugs encapsulated in SEDDS.

## Results

### Avocado polyol physical characterization studies

**Figure 1:**
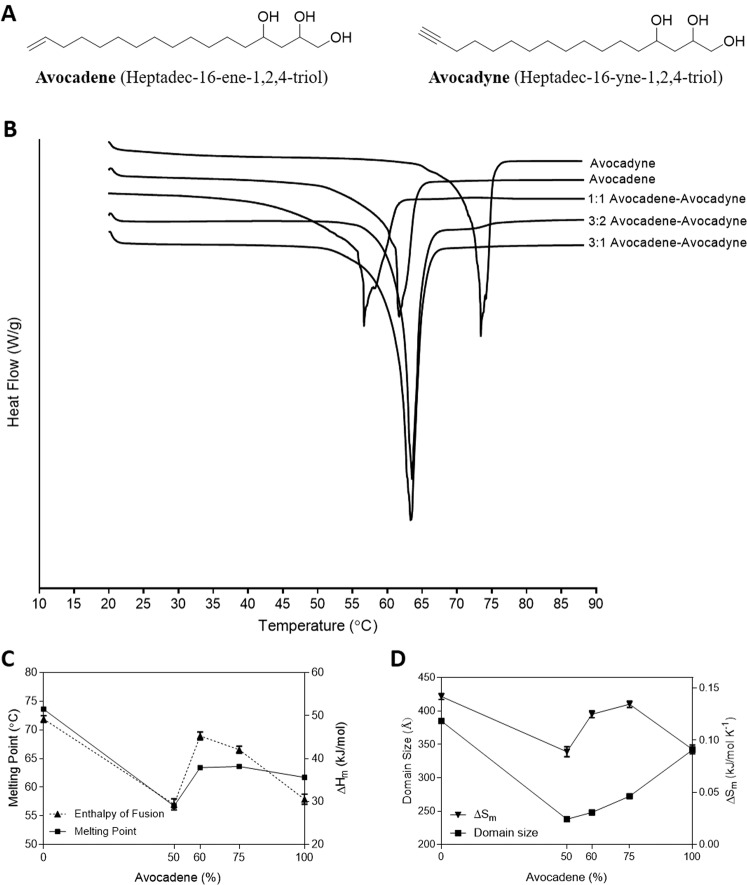
Avocatin B shows eutectic phase behaviour. **(A)** Chemical structures of avocadene and avocadyne. **(B)** Representative DSC melting thermograms for avocadene, avocadyne and their mixtures. Plots have been nudged for illustration. **(C)** Melting temperatures (left Y-axis) and melting enthalpies (right Y-axis) as a function of avocadene and avocadyne composition. **(D)** Domain size (left Y-axis) and melting entropy (right Y-axis), ΔS_m_ as a function of avocadene and avocadyne composition. For (**C,D**) values are means ± SD from three independent experiments.

Purification of avocado seed ethyl acetate extracts yielded a fraction pure in avocadene and avocadyne (Fig. 1A) at a 1:1 (mol:mol) ratio (AVO). Commercially available standards of pure avocadene and avocadyne were purchased for physical characterization studies and also used to generate samples with a 3:1 or 3:2 molar ratio of avocadene:avocadyne. DSC showed avocadyne had a significantly higher melting point than avocadene, as previously reported by Kashman *et al*. who used a Fisher-Johns apparatus^1^ (Fig. 1B). Interestingly, AVO had the lowest peak melting point suggesting that it may be a eutectic composition of avocadene and avocadyne (Fig. 1C). A eutectic composition refers to a binary mixture of two structurally distinct solids that exhibit a depressed melting point relative to either pure component^20^. Importantly, these trends were unaltered when melting onset temperatures for all avocado polyol samples were assessed (data not shown). Furthermore, AVO exhibits a lower value for ΔS_m_ (the ratio of melting enthalpy (ΔH_m_) to the melting point (T_m_) referred to as entropy of melting) compared to pure avocadyne or 3:1 or 3:2 mixtures (Fig. 1D). Collectively, the lower melting point and entropy of AVO in comparison to the other avocado polyol samples is suggestive of a more disordered or liquid-like crystal. Eutectic mixtures identified in other research areas also have low melting points and entropies which is explained by significant differences in intermolecular interactions that introduce greater molecular mobility or disorder^21–23^.

X-ray powder diffraction was performed to determine the molecular arrangement of avocado polyol crystal structures. Measurements in the small and wide angle X-ray scattering regions (SAXS and WAXS, respectively) provide information on the longitudinal and lateral packing of molecules in a crystalline structure, respectively^24^. Diffraction patterns for all avocado polyols in the SAXS region displayed a lamellar crystal arrangement of similar size with characteristic reflections associated with the [001] molecular plane and its higher orders, i.e., [002], [003], [004], etc., evident at the expected ratio of spacings (1:1/2:1/3:1/4:1, and so on) (Supplementary Fig. 3A–E). However, avocadyne lamellae were generally larger and two species of 31 and 40 Angstroms were evident in the [001] plane suggesting the presence of two polymorphs or two geometric isomers. The WAXS patterns for avocadene and mixtures of avocadene and avocadyne were very similar, suggesting that the long hydrocarbon chains of the fatty alcohols pack laterally in similar fashion or in the same polymorphic form. Avocadyne however, displayed a different WAXS patterns than the rest of the samples, suggesting a different unit cell structure. Indexing and unit cell identification were not attempted due to concerns over crystal purity. The observed eutectic behaviour of AVO was attributable to its domain size (i.e., the persistence length and the physical thickness of the nanocrystals in the [001] crystal planes’ direction). This analysis showed that the domain size of AVO was noticeably smaller compared to all other avocado polyol samples whereas avocadyne exhibited the largest domain size (Fig. 1D). The relationship between small domain sizes and lower melting points has widely been reported for both eutectic^25,26^ and monotectic systems^27^. Furthermore, our XRD analysis on structurally related odd-numbered carbon lipids (i.e., 1-heptadecanol, heptadecanoic acid and 16-heptadecynoic acid) showed larger lamellae (SAXS diffraction peaks), greater domain sizes, and different lateral packing of hydrocarbon chains (WAXS diffraction peaks) compared to avocado polyols (Supplementary Fig. 4A–C).

### SEDDS development and characterization

Our previous work has shown that AVO and its individual constituents have limited aqueous solubility. Thus, we evaluated if existing O/W self-emulsifying systems, commonly used in food and pharmaceutical formulations^18,28,29^, could be used to easily incorporate avocado polyols. Supplementary Table 1 shows a list of formulations that were tested for self-emulsification. Of the SEDDS tested, NeobeeM5 or coconut oil in combination with Tween 80 or Cremophor EL (CrEL), at an oil/surfactant ratio of 1:1, were the most suitable SEDDS, when diluted 10 folds in phophate-buffered saline (PBS) (Supplementary Fig. 5). Higher weight ratios of NeobeeM5 to Tween 80 or CrEL (greater than 3:1) generally resulted in larger droplet sizes with higher polydispersity (Supplementary Fig. 5). The cytotoxicity of 1:1 NeobeeM5—CrEL SEDDS (mean droplet diameter of ~80 nm) compared to NeobeeM5—Tween 80 SEDDS (mean droplet diameter of ~200 nm) was significantly greater in AML (OCI-AML-2) and non-AML cell lines (INS-1 (832/13), Caco-2, and HepG2) (Supplementary Fig. 6). As such, only 1:1 NeobeeM5—Tween 80 was further studied as the chosen SEDDS system.

**Figure 2:**
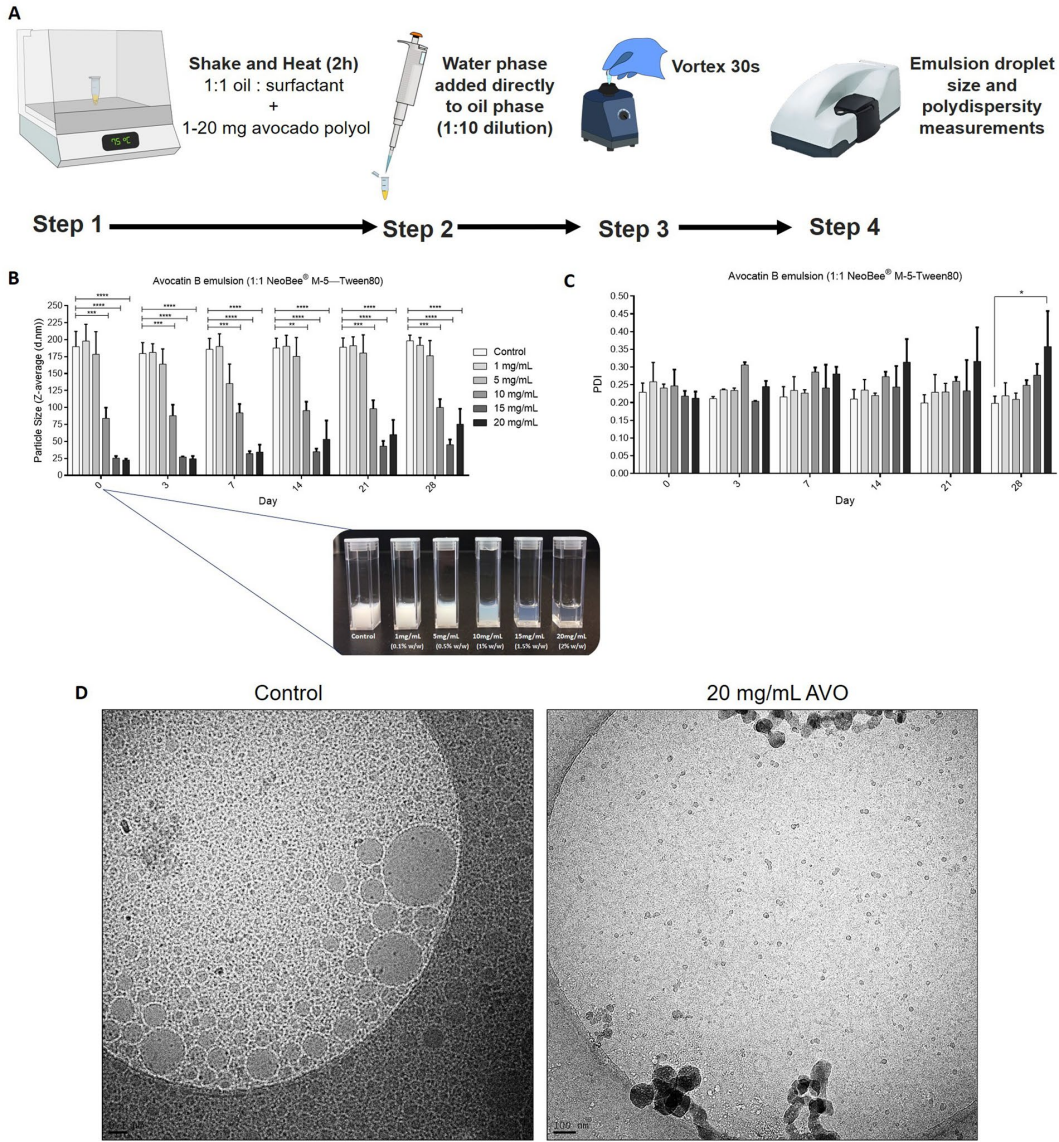
Avocatin B reduces droplet size of a MCT oil— polysorbate 80 based SEDDS. **(A)** SEDDs method of preparation. **(B)** Effect of avocatin B concentration on average hydrodynamic diameter (Z-average) of NeoBee—Tween 80 SEDDs over time. Inset: visual appearance of control (blank SEDDS) and avocatin B containing SEDDS on day 0. **(C)** Polydispersity index of SEDDS described in (**B**). Values are means ± SEM of three independent experiments. **(D)** Cryo-TEM images of control (blank SEDDS) and 20 mg/mL avocatin B containing SEDDs. Scale bar represents 100 nm.

Avocado polyols self-assembled at the O/W interface of the chosen SEDDS. Avocado polyols (1–20 mg) were pre-dissolved in 100 µL of the oil/surfactant phase by heating to 75 °C for 2 hr, after which 900 µL water phase (PBS) was added directly to the oil phase and vortexed for 30 sec (Fig. 2A). This method of preparation was necessary to produce consistent emulsion droplet sizes; another method where hot oil phase was added to PBS under constant stirring did not produce consistent results. When 1–2% (w/w) AVO was incorporated into SEDDS, significant reduction in turbidity (as characterized by an increase in transparency) and mean droplet size were observed where 2% (w/w) AVO reduced SEDDS droplet diameter below 25 nm (Fig. 2B). Ambient temperature polydispersity and droplet size measurements increased for 1.5–2% (w/w) AVO containing SEDDS over four weeks compared to control SEDDS (Fig. 2C). Cryo-transmission electron microscopy (TEM) images of the control and 2% (w/w) AVO emulsions highlighted the drastic reduction in mean droplet size caused by AVO (Fig. 2D). Similarly, AVO significantly reduced droplet diameter of 1:1 coconut oil—Tween 80 SEDDS (Supplementary Fig. 7A,B) and 1:1 NeobeeM5—CrEL SEDDS (Supplementary Fig. 7C,D) in a concentration dependent manner. Hydrophilic co-surfactants like glycerol, propylene glycol and ethanol have also been reported to penetrate the interfacial film and decrease droplet size of O/W emulsions^15,16,30^; however, no such effects were observed in the chosen SEDDS system (data not shown). Collectively, this highlights AVO as a novel and effective co-surfactant with potential to be utilized in other delivery systems.

**Figure 3:**
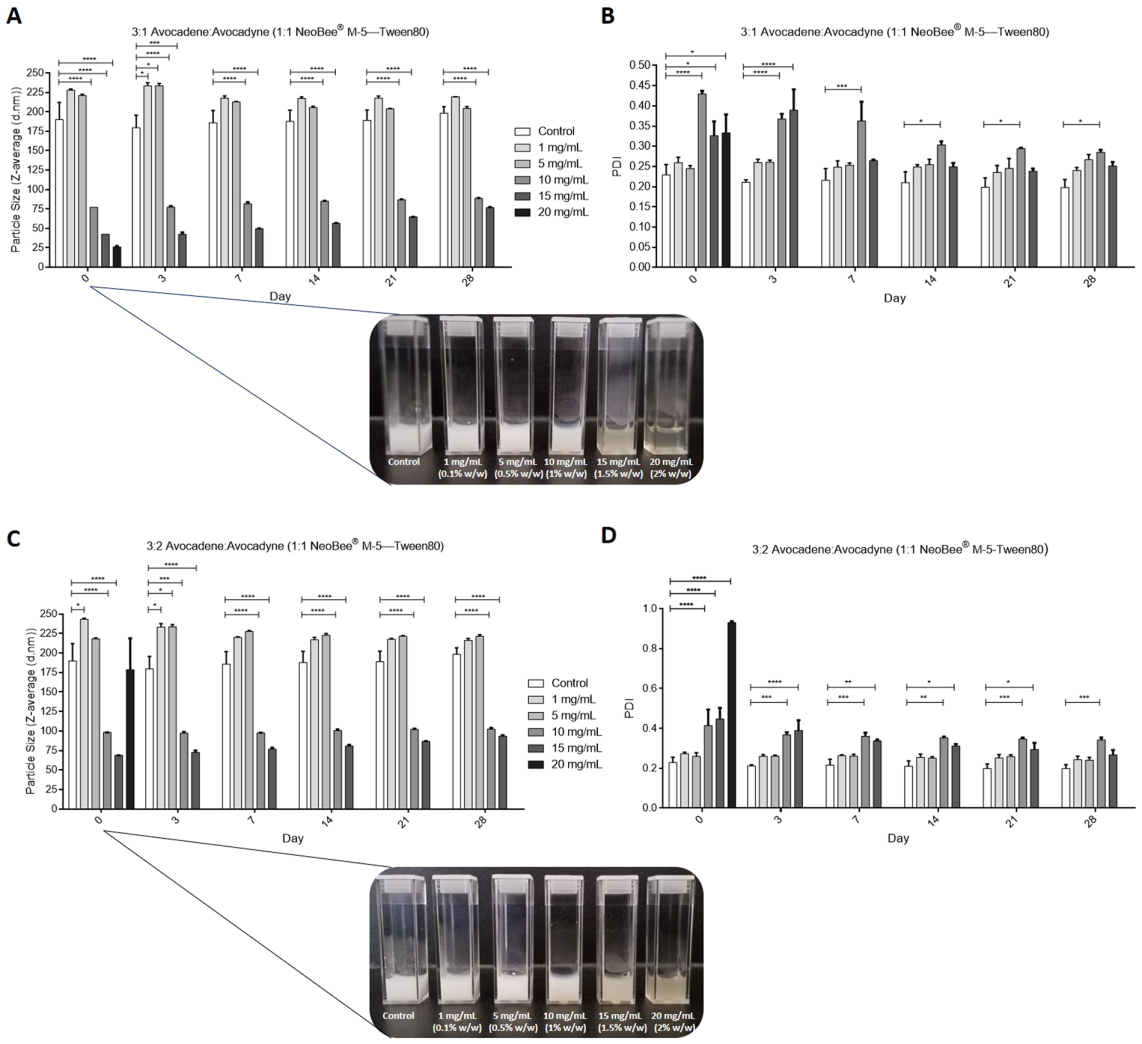
The ratio of avocadene and avocadyne dictates SEDDS droplet size and stability. **(A)** Effect of 3:1 avocadene-avocadyne concentration on average hydrodynamic diameter (Z-average) of NeoBee—Tween 80 SEDDS over time. Inset: visual appearance of control (blank SEDDs) and 3:1 avocadene-avocadyne containing SEDDS on day 0. **(B)** Polydispersity index of SEDDS described in (**A**). **(C)** Effect of 3:2 avocadene-avocadyne concentration on Z-average of NeoBee—Tween 80 SEDDS over time. Inset: visual appearance of control (blank SEDDs) and 3:2 avocadene-avocadyne containing SEDDs on day 0. **(D)** Polydispersity index of SEDDs described in (**C**). For **A**–**D**, values are means ± SEM of three independent experiments. Note: size and polydispersity data for both 3:1 and 3:2 avocadene-avocadyne at a concentration of 20 mg/mL are missing on day 3 and onwards due to emulsion destabilization.

Choice of raw material (avocado pulp or seed from different cultivars) and differences in extraction methods often result in the purification of variable ratios of avocadene and avocadyne^3^. Since AVO is a 1:1 ratio of avocadene and avocadyne; 3:1 and 3:2 ratios were generated using pure avocadene and avocadyne and incorporated into SEDDS to determine their self-emulsifying properties. Similar to AVO, 3:1 avocadene—avocadyne SEDDS formed mean droplet sizes ~25 nm at 2% (w/w); however, this emulsion destabilized after 24 hr (Fig. 3A,B). In contrast, 3:2 avocadene—avocadyne SEDDS exhibited larger droplet sizes at concentrations between 0.1–0.15% (w/w) compared to AVO or 3:1 avocadene—avocadyne SEDDS (Fig. 3C,D). Together, this data highlights the importance of avocadene: avocadyne ratios for optimal incorporation into SEDDS.

**Figure 4:**
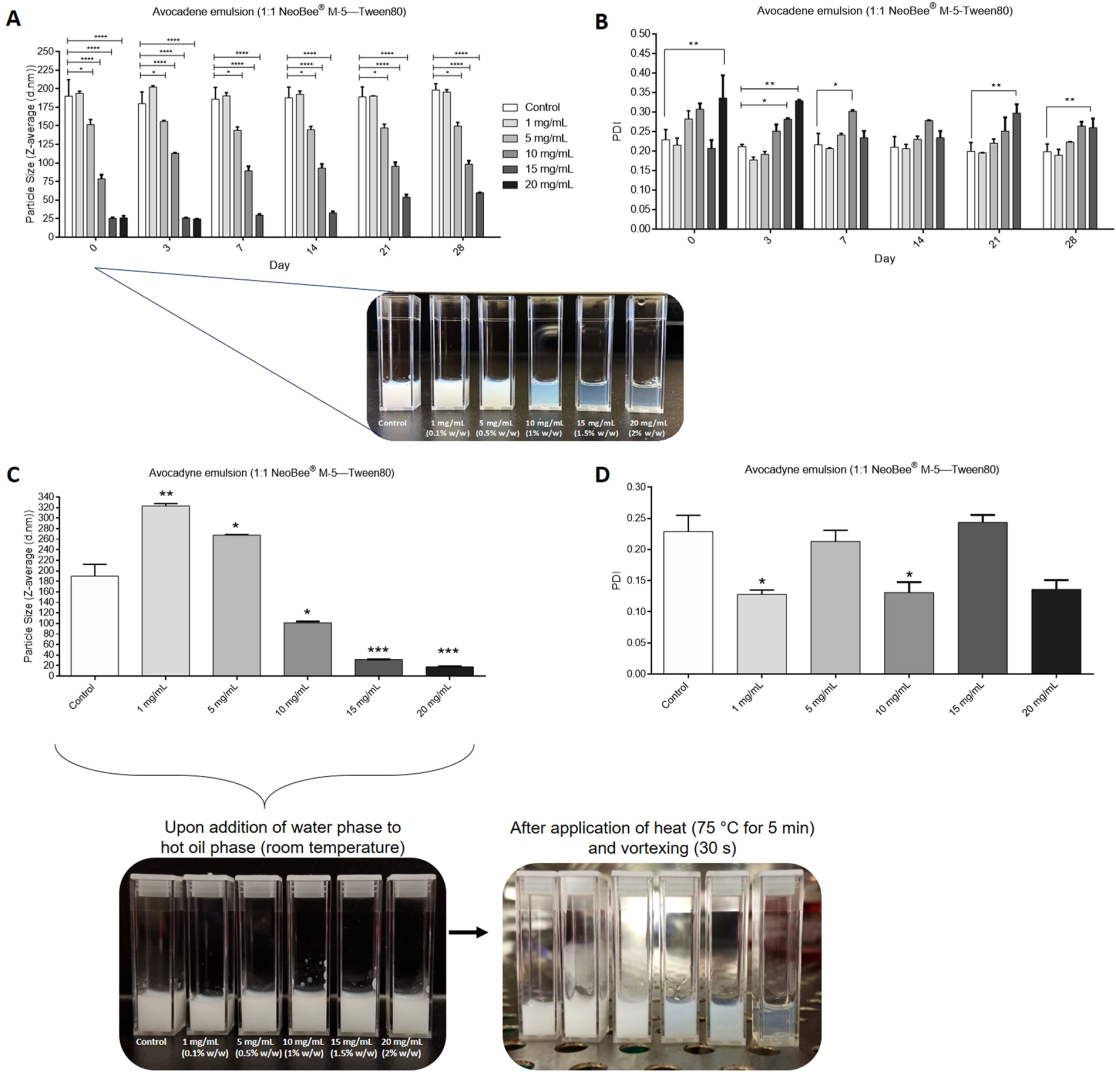
Avocadene and avocadyne behave differently in SEDDS. **(A)** Effect of avocadene concentration on average hydrodynamic diameter (Z-average) of NeoBee—Tween 80 SEDDs over time. Inset: visual appearance of control (blank SEDDS) and avocadene containing SEDDS on day 0. **(B)** Polydispersity index of SEDDS described in (**A**). **(C)** Effect of avocadyne concentration on Z-average of NeoBee—Tween 80 SEDDS when final emulsions are heated for 3–5 min and droplet size is measured at 37 °C. Inset: visual appearance of control (blank SEDDs) and avocadyne containing SEDDS before and after application of heat (emulsion formation). **(D)** Polydispersity index of SEDDs described in (**C**). For A–D, values are means ± SEM of three independent experiments. Note: size and polydispersity data for avocadene at a concentration of 20 mg/mL is missing on day 7 and onwards due to emulsion destabilization.

Next, we incorporated pure avocadene or avocadyne into SEDDS to determine their individual effects. Avocadene in SEDDS behaved similar to AVO where 0.5–2% (w/w) avocadene caused a significant reduction in droplet diameter compared to control (Fig. 4A,B). Surprisingly, avocadyne did not exhibit the same behaviour as AVO or avocadene in the chosen SEDDS system and microemulsions did not form when water phase was added to hot oil phase containing 1–20 mg of avocadyne (Fig. 4C,D). However, fine transparent microemulsions for 1–2% (w/w) avocadyne were observed after application of heat (75 °C for 3–5 minutes) and keeping samples at 37 °C.

DSC was also used to evaluate the melting temperatures of avocado polyols when incorporated in only the oil/surfactant phase (at a concentration of 200 mg/mL) of the SEDDS (1:1 NeobeeM5—Tween 80). This analysis also revealed that avocadyne exhibited larger thermal and entropic parameters compared to all other polyol samples (Supplementary Fig. 8A,B). Collectively, differences in crystal structure and packing as well as solubility of avocado polyols in the chosen oil phase are integral factors that impact self-assembly.

### Stability of avocado polyol SEDDS

Investigation on the mechanism of destabilization of avocado polyol SEDDS revealed that a combination of Ostwald ripening and coalescence take place as determined by regression analysis on the cube of mean droplet radius (r^3^) (Ostwald ripening model) and inverse of the square of mean droplet radius (1/r^2^) (coalescence model)^30^ (Supplementary Table 2). Particle size distribution (PSD) analysis over time provided additional insight into the kinetics of the two destabilization mechanisms^31^. Both PSD peak broadening (suggestive of coalescence occurring at a faster rate) and sharpening (suggestive of Ostwald ripening following first order kinetics) was observed over time in a concentration independent manner for all polyol SEDDS (Supplementary Figs. 9A–C and 10A–F). In contrast, further dilution of all the described avocado polyol SEDDS (up to 1000 folds in PBS) slowed droplet size growth significantly (data not shown); a finding widely reported for micro and nano- self-emulsifying systems^32,33^. PBS was chosen as the aqueous phase for all SEDDS characterization studies to ensure no changes to osmotic and pH balance *in vitro* and *in vivo*^34^. Zeta potential values for all SEDDS prepared in PBS at 10–1000 fold dilutions were not significantly different (i.e., less than −6 mV) (data not shown). Thermodynamic stability was assessed for freshly prepared avocado polyol SEDDS using centrifugation and freeze-thaw tests. AVO SEDDS (up to 2% (w/w)) did not flocculate or cream after ultracentrifugation (Supplementary Fig. 9D). AVO SEDDS freeze at −20 °C, but an isotropic emulsion is reformed after thawing at 37 °C (Supplementary Fig. 9E). Bright field microscopy of a six-month aged 2% (w/w) AVO SEDDS showed large crystals (a combination of AVO, oil and surfactant) which disappeared after heating (45 °C) for 3–5 min (Supplementary Fig. 9F) suggesting that a destabilized polyol microemulsion can undergo self-emulsification with heating. Polarized light microscopy confirmed that all avocado polyol SEDDS were isotropic colloidal dispersions void of lamellar liquid crystals (Supplementary Fig. 11), as no birefringence was observed between crossed polarizing plates^35^. These properties were observed for all freshly prepared avocado polyol SEDDS.

### *In vitro* activity of avocado polyol SEDDS

**Figure 5:**
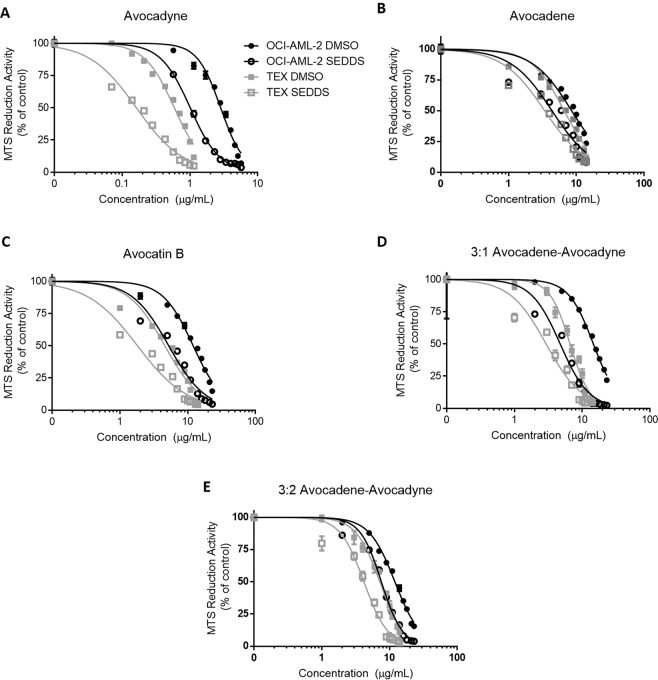
Avocado polyol SEDDS selectively reduce viability of AML cells with enhanced potency compared to DMSO delivery. *In vitro* activity of avocado polyol SEDDS was evaluated in AML cell lines OCI-AML-2 and TEX. Cells were incubated with varying concentrations of avocado polyols dissolved in DMSO or as SEDDS. After 72 hours, cell viability was measured by the MTS assay. For avocadyne **(A)**, avocadene (**B)**, avocatin B **(C)**, and 3:1 avocadene-avocadyne **(D),** 20 mg/mL (2% w/w) SEDDS were freshly prepared and used for serial dilution. For 3:2 avocadene-avocadyne **(E)**, 15 mg/mL (1.5% w/w) SEDDS were freshly prepared and used. Data represent logarithmic transformation of avocado polyol concentrations (µg/mL) and cell viability data that was fit to a nonlinear four-parameter logistic curve to determine inhibitory concentration 50 (IC50). All data represents mean ± SEM from three independent experiments performed in triplicate.

Previously, it has been reported that AVO induces mitochondria-mediated death in AML cells^5,6^ where AVO was delivered using a conventional cell culture vehicle of dimethyl sulfoxide (DMSO). Solubility of long aliphatic chain avocado polyols in DMSO is likely a limiting factor for *in vitro* cellular delivery, thus we directly compared the cytotoxicity of avocado polyols in AML cells when delivered using DMSO or SEDDS (Fig. 5A–E; Table 1). All avocado polyols delivered in SEDDS lowered AML cell viability with doubled potency compared to DMSO delivery.

**Table 1:** IC50 values of avocado polyols delivered either in DMSO or SEDDS to OCI-AML-2 or TEX cells.

	OCI-AML-2		TEX	
	IC50 in DMSO (µg/mL)	IC50 in SEDDS (µg/mL)	IC50 in DMSO (µg/mL)	IC50 in SEDDS (µg/mL)
Avocadyne	2.84 ± 0.18^a^	1.02 ± 0.05^b^	0.59 ± 0.02^a^	0.16 ± 0.01^a^
Avocadene	8.49 ± 0.41^a^	4.36 ± 0.12^b^	6.62 ± 0.33^a^	3.42 ± 0.18^b^
Avocatin B	12.05 ± 0.93^a^	5.24 ± 0.19^b^	4.59 ± 0.18^a^	1.81 ± 0.04^b^
3:1 avocadene-avocadyne	14.59 ± 0.55^a^	4.73 ± 0.25^b^	6.60 ± 0.63^a^	2.93 ± 0.40^b^
3:2 avocadene-avocadyne	12.01 ± 0.45^a^	7.58 ± 0.22^b^	7.18 ± 0.72^a^	4.18 ± 0.42^b^

IC50 was calculated using non-linear regression model (logarithmic inhibitor vs. normalized response-variable slope).

Different superscript letters within each row for each cell line indicates significant differences (P < 0.0001) between DMSO and SEDDS delivery, two-way ANOVA with Sidak’s post hoc test (n = 3).

**Figure 6:**
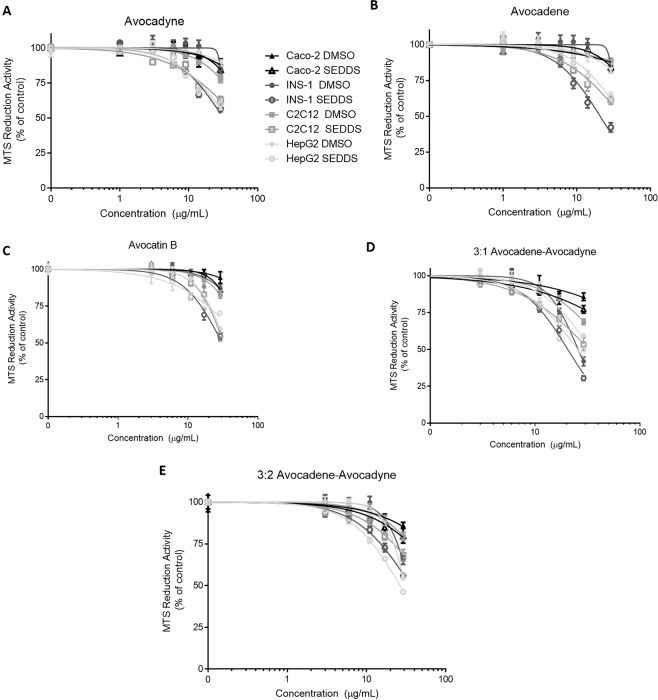
Avocado polyols exert less toxicity in non-AML cell lines when delivered in either DMSO or SEDDS. *In vitro* cytotoxicity of avocado polyols was evaluated in non-AML cell lines (Caco-2, INS-1 (832/13), C2C12 myotubes and HepG2). Cell lines were incubated with varying concentrations of avocado polyols dissolved in DMSO or as SEDDS. After 24 hours, cell viability was measured by the MTS assay. For avocadyne **(A)**, avocadene (**B)**, avocatin B **(C)** and 3:1 avocadene-avocadyne **(D),** 20 mg/mL (2% w/w) SEDDS were freshly prepared and used. For 3:2 avocadene-avocadyne **(E),** 15 mg/mL (1.5% w/w) SEDDS were freshly prepared and used. Data fit to nonlinear regression curve as described for Fig. 5. All data represents mean ± SEM from three independent experiments performed in triplicate.

Avocado polyols selectively exert cytotoxicity in AML cells while sparing non-AML cells^5,36^. Hence, we tested the cytotoxicity of avocado polyols (delivered in DMSO or SEDDS) in non-AML, adherent, cells such as INS-1 (832/13), C2C12 myotubes, Caco-2, and HepG2 (Fig. 6A–E). Notably, all avocado polyols delivered in DMSO showed no activity in any of the non-AML cell lines even at high concentration ranges between 15–29 µg/mL. However, avocado polyols delivered in SEDDS at the supraphysiological concentration of 29 µg/mL (>50 µM) showed some reductions in viability in INS-1 (932/13), C2C12 myotubes and HepG2 cells suggesting enhanced delivery. The greater *in vitro* potency of avocado polyol SEDDS in AML cell lines demonstrated here will enable more effective dosing of these compounds in future *in vitro* and *in vivo* studies.

### Pilot *in vivo* pharmacokinetic study

**Figure 7:**
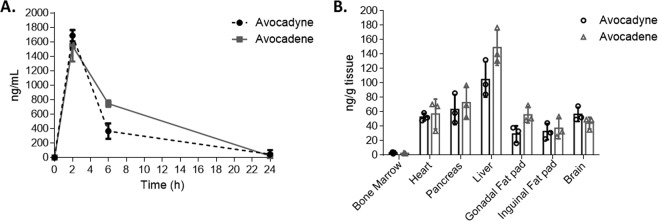
Avocatin B SEDDS show bioavailability and biodistribution in a pilot *in vivo* pharmacokinetic study. Avocatin B SEDDS (2% w/w) was delivered via gavage (100 mg/kg body weight (b.w.)) to 6–8 week old female C57BL/6J mice. **(A)** 50–100 µL blood was collected via tail-snips at 2 hr, 6 hr and endpoint (24 hr). Avocadene and avocadyne were quantified in blood using a validated LC-MS bio-analytical method. **(B)** Tissues (bone marrow, heart, pancreas, liver, gonadal fat pad, inguinal fat pad, and brain) were collected at endpoint for avocadene and avocadyne quantification. Data are shown as mean ± S.D., N = 3 in each group.

The *in vivo* nutritional significance, bioavailability, and biodistribution of long chain fatty alcohols is not well studied^3,37^. The pharmacokinetics of a 100 mg/kg (body weight (b.w.)) oral bolus dose containing 20 mg/mL AVO SEDDS was examined in mice. Non-compartmental pharmacokinetic analysis showed that the maximum concentration (Cmax) of avocadyne and avocadene in whole blood was 1687.90 and 1544.83 ng/mL, respectively (Table 2). Despite similar maximal plasma concentrations (Fig. 7A), exposure to avocadene seemed greater than avocadyne due to its higher area under the curve (AUC_0–24h_), however more time points and are needed to decipher statistically significant differences in all pharmacokinetic parameters. Furthermore, avocadyne and avocadene were detectable in various metabolically active tissues 24 h from oral administration, accumulating most in the liver and pancreas (Fig. 7B). This pilot pharmacokinetic study also allowed for the development and validation of a bioanalytical method that has desirable selectivity, linearity, extraction recovery, accuracy and precision for the quantitation of AVO in mouse whole blood and tissue matrices (Supplementary Table 3).

**Table 2:** Avocatin B non-compartmental pilot pharmacokinetic analysis. Data are shown as mean ± S.D., N = 3 in each group.

	Avocadene	Avocadyne
	Mean ± S.D.	Mean ± S.D.
Cmax (ng/mL)	1687.90 ± 79.08^a^	1544.83 ± 216.37^a^
Tmax (h)	2.00 ± 0.00	2.00 ± 0.00
Kel (h^−1^)	0.18 ± 0.06^a^	0.20 ± 0.03^a^
t_1/2_ (h)	4.37 ± 1.88^a^	3.55 ± 0.50^a^
AUC_0_t_ (ng/ml*h)	9499.48 ± 1243.22^a^	13032.37 ± 838.88^b^
AUC_0_inf_ (ng/mL*h)	9071.26 ± 3480.83^a^	9868.07 ± 2253.16^a^

Cmax denotes maximum concentration of bioactive in blood; Tmax denotes time at which Cmax occurs; Kel denotes elimination rate constant; t1/2 denotes half-life of bioactive in blood; AUC0_t denotes area-under-the-curve (AUC) from time 0–24 hr; AUC0_inf denotes AUC from time 0 to infinity. Different superscript letters within each row indicates significant differences (P < 0.05) between avocadene and avocadyne, two-tailed Student’s t-test (n = 3).

Our previous work reports the positive effects of AVO in a mouse model of diet-induced obesity and utilized the AVO SEDDS described in this work to deliver an oral dose of 100 mg AVO/kg (body weight (b.w.)) twice weekly for five weeks to obese mice on a high fat diet^10^. In a prior unpublished study, we utilized the same AVO SEDDS to deliver 100 mg AVO/kg (b.w.) twice weekly for 13 weeks to mice on standard and high fat diet. In both studies, repeated oral dosing of AVO SEDDS for a prolonged period of time in lean and obese mice showed no adverse effects as assessed by onset of physical abnormalities, excessive weight loss and significant changes in endpoint complete blood count data. Although more tolerability data (changes in liver, muscle, kidney and heart enzymes) is required to completely evaluate the safety of AVO SEDDS, it is likely the low concentrations of excipients in the SEDDS are advantageous from a safety perspective.

### Other applications of avocado polyol SEDDS

**Figure 8:**
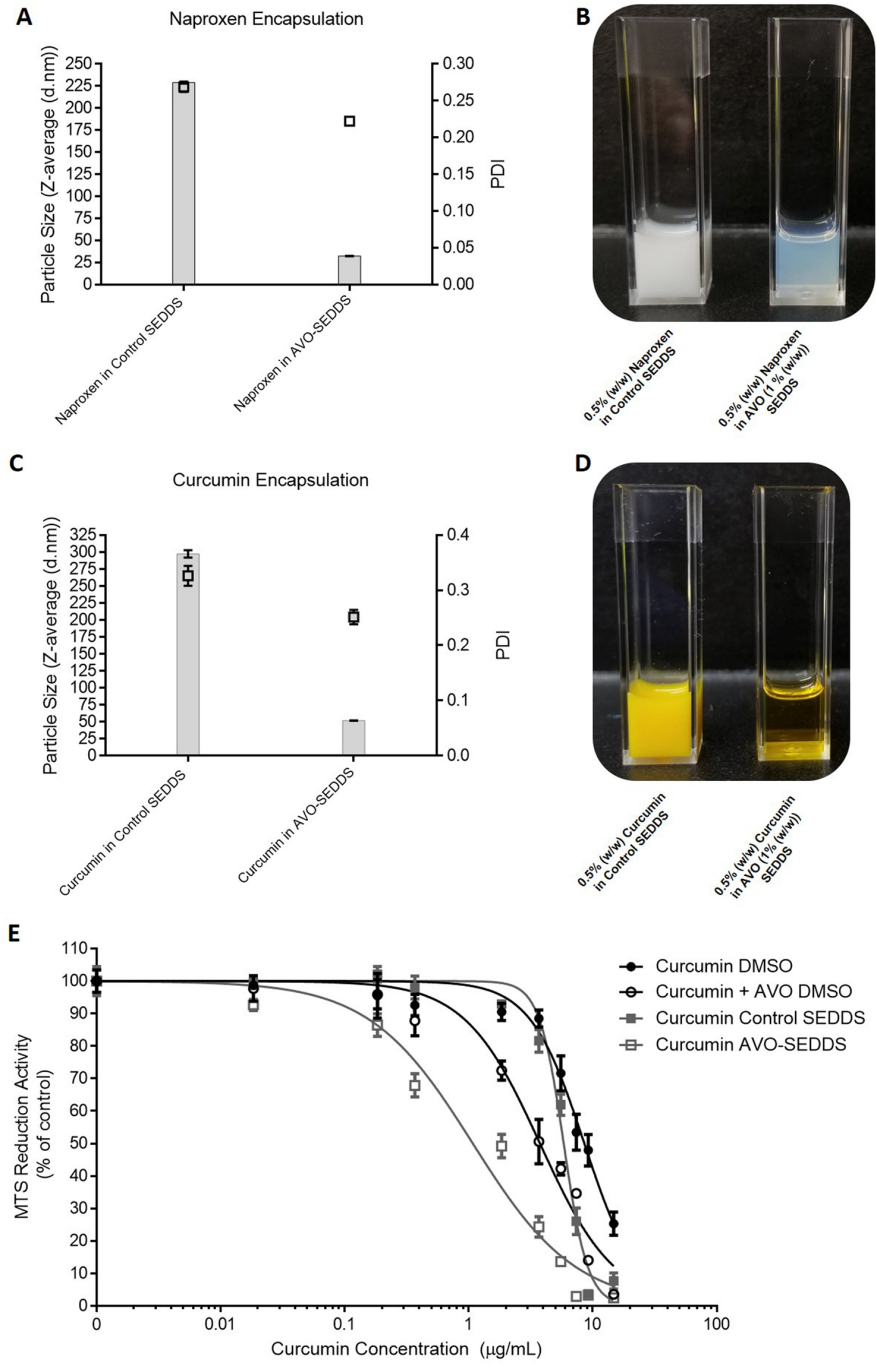
AVO improves encapsulation of poorly water soluble drugs. **(A)** Histograms represent Z-average (left Y-axis) of Naproxen (0.5% (w/w)) encapsulated in NeoBee—Tween 80 SEDDS (control SEDDS) and AVO (1% (w/w)) SEDDS. Symbol represents PDI (right Y-axis) **(B)** Visual appearance of SEDDS described in (**A**). **(C)** Z-average and PDI of curcumin (0.5% (w/w)) in control or AVO (1% (w/w)) SEDDS. **(D)** Visual appearance of SEDDS described in (**C**). **(E)** *In vitro* cytotoxicity of curcumin formulated in DMSO or SEDDS with or without AVO was evaluated in OCI-AML-2 cells. Data fit to nonlinear regression curve described for Fig. 5. For (**A**,**C**) data represents mean ± SEM of two independent experiments. For (**E**), data represents mean ± SEM from three independent experiments performed in triplicate.

SEDDS are often commercialized in solid dosage forms where the oil phase and drugs are adsorbed onto a solid carrier and used to produce tablets, capsules etc., for oral consumption^29^. The oil phase for both control and AVO SEDDS adsorbed easily onto the solid carrier neusilin^38^. These solid-SEDDS self-assembled into expected droplet size distributions upon dissolution in water (Supplementary Fig. 12) suggesting the described SEDDS are scalable and suitable for common commercial dosage forms.The ability of AVO to enhance encapsulation of poorly water soluble compounds such as naproxen and curcumin was also tested. In a previous study, naproxen (3 mg/mL) encapsulated in 1:1 NeoBeeM5—Tween 80 SEDDS had a mean droplet size of 240 nm^28^; however, the method of SEDDS preparation was different than described herein. In this study, 5 mg/mL naproxen encapsulated in AVO-SEDDS was observed to have a mean droplet size of 32 nm compared to naproxen in control SEDDS which was 229 nm (Fig. 8A,B). Similarly, 5 mg/mL curcumin encapsulated in AVO-SEDDS showed a mean droplet size of 52 nm compared to 297 nm in control SEDDS (Fig. 8C,D). Both naproxen and curcumin control and AVO SEDDS destabilized over the course of 3 days; however, higher dilutions (above 1:100) slowed this effect to several weeks (data not shown). Finally, curcumin has an established *in vitro* bioactivity profile in AML cells^39^; thus, we delivered curcumin to OCI-AML-2 cells in a DMSO or SEDDS formulation (Fig. 8E). Expectedly, curcumin in AVO-SEDDS had greater potency compared to curcumin/AVO in DMSO or curcumin in control SEDDS (Fig. 8E and Table 3).

**Table 3:** IC50 values of curcumin delivered via DMSO or in SEDDS to OCI-AML-2 cells. IC50 was calculated using non-linear regression model (logarithmic inhibitor vs. normalized response-variable slope).

	DMSO (µg/mL)	SEDDS (µg/mL)
Curcumin	8.55 ± 0.10^a^	5.83 ± 0.19^b^
Curcumin + AVO	3.75 ± 0.18^a^	1.08 ± 0.13^b^

Different superscript letters within each row indicate significant differences (*P *< 0.0001) between treatments, two-way ANOVA with Sidak’s post hoc test (n = 3).

## Discussion

Avocado derived avocadene and avocadyne are prevalent in the cosmetic, agricultural and food industries. Their diverse medicinal properties warrant additional research that evaluates these natural compounds as novel therapies for human diseases. This study highlights strong links between the physical properties of avocado polyols extracted from avocado seeds to: i) their behaviour in SEDDS, ii) *in vitro* bioactivity, and iii) *in vivo* pharmacokinetics. The physical characterization of avocadene, avocadyne and their binary mixtures through DSC and XRD reveals valuable new data providing key insights for future large-scale formulation studies. The eutectic phase behaviour of AVO is advantageous for its successful incorporation into simple, low-energy, bio-compatible SEDDS. Eutectic mixtures are shown to improve drug dissolution/solubility and bioavailability^40–42^; however, these systems employ structurally unrelated molecules (e.g. drug and excipients) to form eutectics. In contrast, avocadene and avocadyne are two structurally similar natural compounds that form a eutectic mixture at a molar ratio of 1:1 which translates to their efficient incorporation into a microemulsion-based delivery system. Mechanisms of eutectic formation are generally attributed to differences in i) molecular size and geometry, ii) polymorphic state, and iii) external fields (cooling and shear rates), which collectively leads to the formation of a more disordered crystal packing^25^. The XRD data presented here confirmed that molecular size and potentially polymorphic state contribute to the eutectic formation seen in AVO.

The incorporation of small amounts of AVO into SEDDS resulted in drastic reductions in droplet diameter without adding energy or a co-surfactant. This is strongly indicative of AVO self-assembling at the oil-water interface altering the interface curvature and film flexibility. The co-surfactant effects of avocado polyols in MCT oil—high HLB surfactant SEDDS correlated well with their physical properties (notably melting temperatures, enthalpies and entropy as well as crystal structure and thickness). Avocadene was demonstrated to be more compatible in SEDDS than avocadyne. The heat sensitive nature of avocadyne SEDDS was an unexpected finding which requires further evaluation to link its unique physical properties to extent of self-assembly. Furthermore, all avocado polyol SEDDS showed signs of destabilization at concentrations greater than or equal to 2% (w/w) suggesting that higher polyol concentrations may penetrate the interfacial film in such a way that pushes the spontaneous curvature of the surfactant film past the hydrophilic—lipophilic balance point^30,43^.

All avocado polyol SEDDS showed enhanced *in vitro* potency compared to DMSO delivery in AML cell lines, while maintaining selective toxicity. We hypothesize SEDDS enabled increased accumulation of polyols in molecular targets (predominantly mitochondria) compared to DMSO. However, further *in vitro* studies that test the solubility, release profile^44^, and cellular uptake^45^ of avocado polyols in SEDDS versus conventional cell culture solvents are required to elucidate the mechanisms by which SEDDS increase *in vitro* potency. Since the absorption and cellular uptake of long chain fatty alcohols in biological systems is not well understood^37,46^, the pharmacokinetic data presented in this work provides impetus for further research on avocado polyol cellular uptake and absorption. While promising pharmacokinetics and biodistribution was obtained, a full-scale pharmacokinetic study (a minimum of 6–8 time points) is required to deduce absolute bioavailability of AVO delivered in SEDDS (ratio of AUCs obtained from oral and intravenous bolus doses of AVO SEDDS) and generate kinetic biodistribution data in all key tissues including lungs, skeletal muscle and spleen.

Encapsulation of curcumin in AVO-SEDDS highlights the novel use of AVO as both a bioactive and co-surfactant that improves bioactivity and encapsulation of bioactives. However, further experiments are required to determine if AVO has similar effects when curcumin and other anti-cancer drugs are encapsulated in emulsion-based delivery systems different to the one described here.

Lastly, all experimental data in the present study does not account for the potential effects of stereoisomers of avocadene and avocadyne^47^. Avocado polyols utilized in this study were extracted from avocado seed using column chromatography that cannot resolve all four possible stereoisomers of avocadene from those of avocadyne. The chemical synthesis of all 4 stereoisomers of avocadene and avocadyne has recently been reported^48^ which will allow future work to determine i) which stereoisomers are naturally occurring in the avocado, and ii) how different stereoisomers of avocadene and avocadyne behave in physical and biological systems described in this work.

## Methods

### Avocado polyol extraction, purification and analytical characterization

AVO extraction from avocado seeds and analytical characterization is described in section 1 of Supplementary Information. All experimental studies were carried out according to the regulations of the University of Guelph, Environmental Health and Safety office.

### Differential scanning calorimetry (DSC)

Differential scanning calorimeter was used to determine the melting point and transition enthalpies for avocado polyol powders using Q2000 DSC (TA Instruments, Mississauga, ON, Canada). Four to five mg of solid material were measured and placed and sealed in aluminum pans (Mettler Toledo, 51119871, ON, Canada) and heated from 20 °C to 90 °C at 5 °C/min, under a nitrogen purge (gas flow of 18 ml/min). Peak melting temperatures (°C) and enthalpies (J/g) were determined by integrating the endothermic transition using the linear peak integration method in Universal Analysis 2000 (TA Instruments, New Castle, DE) software. Tangent skim method was also utilized to determine melting onset temperature. Melting temperatures and enthalpies were also determined for avocado polyol powders mixed in oil/surfactant (1:1 NeoBeeM5—Tween 80) at a concentration of 200 mg/mL (final amount loaded was 20–30 µL) using the same DSC parameters as described above.

### X-ray powder diffraction

X-ray powder diffraction experiments are described in section 1 of Supplementary Information.

### Preparation and characterization of emulsions

Formulations tested for SEDDS characteristics consisted of either long or medium chain triglycerides and surfactants/co-surfactants with varying HLB values, at various oil-to-surfactant weight ratios (9:1, 5:1, 1:1) (see Supplementary Table 2 for a detailed list of excipients tested). Each formulation was prepared in 2 ml polypropylene centrifuge tubes, vortexed and heated at 75 °C for 5 minutes. The heated oil was then diluted 10-fold with the immediate addition of water phase (PBS) and vortexed for 30 sec. All formulations were visually analyzed for macroscopic appearance (characterized as clear, opalescent or milky appearance). The mean droplet size (Z-average in nm), polydispersity index (PDI), and zeta potential for each formulation was determined using dynamic light scattering (Zetasizer Nano ZS, Malvern Instruments, Malvern, U.K.). A refractive index of 1.33 was used for the aqueous phase, and refractive indices for each oil/surfactant mix was determined using a table top refractometer (Zeiss Abbe, NY, USA). Droplet size was measured on three independently prepared formulations and averaged from three readings per formulation, whereas zeta potential was analyzed on two independent formulations, with each measurement averaged from four readings per formulation. All measurements were carried out at 25 °C except for avocadyne SEDDs where the zetasizer compartment temperature was set to 37 °C to prevent destabilization.

Formulations chosen for further studies were selected based on their self-emulsification properties, small droplet size (below 300 nm), low PDI (below 0.4), and no requirement for co-surfactants. Formulations of avocado polyol-SEDDS contained 1–20 mg powder of each polyol added to 100 µL of a 1:1 mixture of oil and surfactant which was heated to 75 °C for 2 hr in an incubator (MaxQ 4450, Barnstead/Lab-Line, IL, USA). After dissolution of polyols in the oil phase, 900 µL water phase (PBS) was added to the oil phase and vortexed for 30 s. Visual appearance of fresh polyol emulsions were noted followed by droplet size, PDI and zeta potential measurements on day zero (immediate measurement on fresh emulsion), day 3 and weekly for 4 weeks. Poorly water-soluble drugs naproxen and curcumin were also encapsulated in SEDDS. Briefly, naproxen or curcumin were added to oil phase (1:1 NeoBeeM5—Tween 80) at a concentration of 50 mg/mL with or without 100 mg/mL AVO and heated overnight at 75 °C and then the oil phase was diluted 10 folds with PBS and vortexed for 30 s. Droplet size and PDI were then measured as described above.

### Cryo-transmission electron microscopy

Transmission electron microscopy (TEM) was conducted at the Advanced Imaging Center in the University of Guelph. Approximately 5 μl of emulsion was placed on a carbon grid with a perforated carbon film (Canemco & Marivac, Quebec, Canada). Filter paper was used to blot the excess liquid. The grid was then immediately dipped into liquid ethane and transferred to a cryo-holder for direct observation at −176 °C on a FEI Tecnai G2 F20 energy-filtered Cryo-TEM (FEI Corp., Hillsboro, Oregon, USA) operated at 200 kV in low dose mode, equipped with a Gatan 4k CCD camera (Gatan Inc., Pleasanton, California, USA).

### Optical and polarized light microscopy

Optical and polarized light microscopy was performed using an optical microscope model BX60 (Olympus Optical Co., Tokyo, Japan). Images were captured (using 20× objective lens) with a model DP71 digital camera (Olympus Optical Co., Tokyo, Japan) using the cellSens (version 1.0) software. 20–50 µL of emulsion samples were placed on a microscope slide and micrographs were obtained using either brightfield or polarized light. Polarized light microscopy was performed on emulsions to confirm absence of crystalline phases (i.e., confirm optical isotropy).

### Thermodynamic stability of SEDDs

Emulsion stability was assessed by centrifugation at 21.1 x *g* for 15 min and phase separation, creaming or flocculation was observed. The effect of temperature on SEDDS was also evaluated using freeze-thaw tests where formulations were subject to 6 freezing-heating cycles (−20 °C for 24 hr followed by 37 °C for 24 hr) after which visual observations and droplet size measurements were performed.

### Cell culture and *in vitro* cytotoxicity

Cell culture and *in vitro* cytotoxicity assay methods are described in section 1 of Supplementary Information.

### *In vivo* pilot pharmacokinetic study

*In vivo* study design, procedures and pharmacokinetic data analysis is described in section 1 of Supplementary Information. All animal studies were carried out according to the regulations of the Canadian Council on Animal Care and with the approval of the University of Guelph, Animal Care Committee.

### Preparation of solid-SEDDS

Solid-SEDDS preparation is described in section 1 of Supplementary Information.

### Statistical data analysis

Unless otherwise stated, DSC results are presented as mean ± S.D., droplet size and PDI measurements for SEDDS are presented as mean ± SEM, *in vitro* results are presented as mean ± SEM, and *in vivo* results are presented as mean ± SD. Data were analyzed with GraphPad Prism 6.0 (GraphPad Software, USA) using one or two-way ANOVA with Tukey’s or Dunnett’s post hoc analysis for between group comparisons. Standard student’s t-tests were also used where appropriate. P < 0.05 was accepted as being statistically significant where *p < 0.05, **p < 0.01, ***p < 0.001, ****p < 0.0001.

## Supplementary information

Supplementary information.

## References

[1] Kashman Y, Neeman I, Lifshitz A (1969). New Compounds from Avocado Pear. Tetrahedron.

[2] Neeman I, Lifshitz A, Kashman Y (1969). A New Antibacterial Agent Isolated from Avocado Pear. Isr. J. Chem..

[3] Ahmed N, Smith RW, Henao JJA, Stark KD, Spagnuolo PA (2018). Analytical Method To Detect and Quantify Avocatin B in Hass Avocado Seed and Pulp Matter. J. Nat. Prod..

[4] Bhuyan DJ (2019). The Odyssey of Bioactive Compounds in Avocado (Persea americana) and Their Health Benefits. Antioxidants.

[5] Lee EA (2015). Targeting mitochondria with avocatin B induces selective leukemia cell death. Cancer Res..

[6] Tcheng M, Samudio I, Lee EA, Minden MD, Spagnuolo PA (2017). The mitochondria target drug avocatin B synergizes with induction chemotherapeutics to induce leukemia cell death. Leuk Lymphoma.

[7] Spagnuolo, P. A., Schimmer, A. D. & Lee, E. A. Avocado-derived lipids for use in treating leukemia. United States patent US 20170304251A1. 2017 Oct 26.

[8] Tabe Y (2018). Inhibition of FAO in AML co-cultured with BM adipocytes: Mechanisms of survival and chemosensitization to cytarabine. Sci. Rep..

[9] Wu, Y.-H. et al. Avocado (Persea americana) fruit extract (2R, 4R)−1, 2, 4-trihydroxyheptadec-16-yne inhibits dengue virus replication via upregulation of NF-κB–dependent induction of antiviral interferon responses. *Sci. Rep*. **9** (2019). 10.1038/s41598-018-36714-4 PMC6344542 30674997

[10] Ahmed, N. et al. Avocatin B Protects Against Lipotoxicity and Improves Insulin Sensitivity in Diet-Induced Obesity. *Mol. Nutr. Food Res*. **63** (2019). 10.1002/mnfr.201900688 31609072

[11] Segal, J. & Rosenblat, G. Polyhydroxylated fatty alcohols. United States patent US 20150175933. 2015 Jun 25.

[12] Msika, P., Legrand, J. & Garnier, S. Use of avocado pit for obtaining an avocado oil enriched with alkyl polyols and/or acetylated derivatives thereof. United States patent US 9,4166,333. 2016 Aug 16.

[13] Mcclements DJ (2015). Nanoemulsions versus microemulsions: Terminology, differences, and similarities Nanoemulsions versus microemulsions: terminology, differences, and similarities. R. Soc. Chem..

[14] Nagarajan R, Wang CC (2000). Theory of surfactant aggregation in water/ethylene glycol mixed solvents. Langmuir.

[15] Gradzielski M (2002). Effect of the Cosurfactant Structure on the Bending Elasticity in Nonionic Oil-in-Water Microemulsions. Langmuir.

[16] Garti N, Yaghmur A, Leser ME, Clement V, Watzke HJ (2001). Improved oil solubilization in oil/water food grade microemulsions in the presence of polyols and ethanol. J. Agric. Food Chem..

[17] Morales D, Gutiérrez JM, García-Celma MJ, Solans YC (2003). A study of the relation between bicontinuous microemulsions and oil/water nano-emulsion formation. Langmuir.

[18] Callender SP, Mathews JA, Kobernyk K, Wettig SD (2017). Microemulsion utility in pharmaceuticals: Implications for multi-drug delivery. Int. J. Pharm..

[19] Solans, C. & Kunieda, H. Industrial Applications of Microemulsions. Surfactant Science, 123–147. (CRC Press, 1997).

[20] Timms RE (1984). Phase behaviour of fats and their mixtures. Prog. Lipid Res..

[21] Peyronel F, Quinn B, Marangoni AG, Pink DA (2014). Ultra small angle x-ray scattering for pure tristearin and tripalmitin: Model predictions and experimental results. Food Biophys..

[22] Hyun JK, Jong HK, Sung HY, Chul SS (2005). Eutectic formation analysis of amino acid mixtures using molecular dynamics simulations. Biotechnol. Prog..

[23] Liu Y (2018). Natural Deep Eutectic Solvents: Properties, Applications, and Perspectives. J. Nat. Prod..

[24] Cullity, B. D. Elements of X-ray Diffraction. 1–136. (Addison-Wesley Publishing, 1956).

[25] Pizzirusso A, Peyronel F, Co ED, Marangoni AG, Milano G (2018). Molecular Insights into the Eutectic Tripalmitin/Tristearin Binary System. J. Am. Chem. Soc..

[26] Marangoni, A. G. & Pink, D. *Edible Nanostructures: A Bottom-up Approach*. (Royal Society of Chemistry, 2015).

[27] Pang YX, Bao X (2002). Aluminium oxide nanoparticles prepared by water-in-oil microemulsions. J. Mater. Chem..

[28] Buyukozturk F, Benneyan JC, Carrier RL (2010). Impact of emulsion-based drug delivery systems on intestinal permeability and drug release kinetics. J. Control. release.

[29] Nardin, I. & Köllner, S. Successful development of oral SEDDS: Screening of excipients from the industrial point of view. *Adv. Drug Deliv. Rev*. (2018). 10.1016/j.addr.2018.10.014 30414496

[30] Wooster TJ, Labbett D, Sanguansri P, Andrews H (2016). Impact of microemulsion inspired approaches on the formation and destabilisation mechanisms of triglyceride nanoemulsions. Soft Matter.

[31] Nazarzadeh E, Anthonypillai T, Sajjadi S (2013). On the growth mechanisms of nanoemulsions. J. Colloid Interface Sci..

[32] Saberi AH, Fang Y, McClements DJ (2013). Fabrication of vitamin E-enriched nanoemulsions by spontaneous emulsification: Effect of propylene glycol and ethanol on formation, stability, and properties. Food Res. Int..

[33] Komaiko JS, McClements DJ (2016). Formation of food‐grade nanoemulsions using low‐energy preparation methods: A review of available methods. Compr. Rev. Food Sci. Food Saf..

[34] Gad SC, Cassidy CD, Aubert N, Spainhour B, Robbe H (2006). Nonclinical vehicle use in studies by multiple routes in multiple species. Int. J. Toxicol..

[35] Friberg SE (1990). Micelles, microemulsions, liquid crystals, and the structure of stratum corneum lipids. J Soc Cosmet Chem.

[36] Samudio I (2010). Pharmacologic inhibition of fatty acid oxidation sensitizes human leukemia cells to apoptosis induction. J Clin Invest.

[37] Hargrove JL, Greenspan P, Hartle DK (2004). Nutritional significance and metabolism of very long chain fatty alcohols and acids from dietary waxes. Exp Biol Med.

[38] Gumaste SG, Freire BOS, Serajuddin ATM (2017). Development of solid SEDDS, VI: effect of precoating of Neusilin® US2 with PVP on drug release from adsorbed self-emulsifying lipid-based formulations. Eur. J. Pharm. Sci..

[39] Rao J (2011). Curcumin reduces expression of Bcl-2, leading to apoptosis in daunorubicin-insensitive CD34+ acute myeloid leukemia cell lines and primary sorted CD34+ acute myeloid leukemia cells. J. Transl. Med..

[40] Figueirêdo CBM (2017). Enhancement of dissolution rate through eutectic mixture and solid solution of posaconazole and benznidazole. Int. J. Pharm..

[41] Wang W (2017). Microemulsions based on paeonol-menthol eutectic mixture for enhanced transdermal delivery: formulation development and *in vitro* evaluation. Artif. cells, nanomedicine, Biotechnol..

[42] Aroso IM (2016). Dissolution enhancement of active pharmaceutical ingredients by therapeutic deep eutectic systems. Eur. J. Pharm. Biopharm..

[43] Kabalnov A, Wennerström H (2002). Macroemulsion Stability: The Oriented Wedge Theory Revisited. Langmuir.

[44] Shen J, Burgess DJ (2013). *In vitro* dissolution testing strategies for nanoparticulate drug delivery systems: Recent developments and challenges. Drug Deliv. Transl. Res..

[45] Mateus A (2017). Intracellular drug bioavailability: A new predictor of system dependent drug disposition. Sci. Rep..

[46] Murota K (2001). Influence of fatty alcohol and other fatty acid derivatives on fatty acid uptake into rat intestinal epithelial cells. Lipids.

[47] Sugiyama T, Sato A, Yamashita K (1982). Synthesis of all four stereoisomers of antibacterial component of avocado. Agric. Biol. Chem..

[48] Cunha, V. L. S., Liu, X., Lowary, T. L. & O’Doherty, G. A. De Novo Asymmetric Synthesis of Avocadyne, Avocadene and Avocadane Stereoisomers. *J. Org. Chem*. (2019). 10.1021/acs.joc.9b02391 31647231

