# Supplementary material for: Avocado-derived polyols for use as novel co-surfactants in low energy self-emulsifying microemulsions
Source: Sci Rep. 2020 Mar 27;10:5566. doi: 10.1038/s41598-020-62334-y (PMC7101315; doi:10.1038/s41598-020-62334-y)
Supplement: Supplementary file 1 — Supplementary information. [file 41598_2020_62334_MOESM1_ESM.docx]

**SUPPLEMENTARY INFORMATION**

**Avocado-derived polyols for use as novel co-surfactants in low energy self-emulsifying microemulsions**

Nawaz Ahmed^1 ѱ^, Behnoush Kermanshahi^1 ѱ^, Saeed M. Ghazani^1^, Katrina Tait^1^_,_ Matthew Tcheng^1^, Alessia Roma^1^, Shannon P. Callender^2^, Richard W. Smith^3^, William Tam^4^, Shawn D. Wettig^2,5^, Michael A. Rogers^1^_,_ Alejandro G. Marangoni^1^, Paul A. Spagnuolo^1^*

^1^ Department of Food Science, University of Guelph, Guelph, Ontario, N1G 2WI, Canada

^2^ School of Pharmacy, University of Waterloo, Waterloo, Ontario N2L3G1, Canada

^3^ University of Waterloo Mass Spectrometry Facility, Department of Chemistry, 200 University Avenue West, Waterloo, ON, N2L 3G1, Canada

^4^ Guelph-Waterloo Centre for Graduate Work in Chemistry and Biochemistry, Department of Chemistry, University of Guelph, Guelph, Ontario, N1G 2W1, Canada.

^5^Waterloo Institute for Nanotechnology, University of Waterloo, 200 University Ave. W., Waterloo, Ontario N2L3G1, Canada

^ѱ^ Authors contributed equally

* Paul A. Spagnuolo, Ph.D.

Associate Professor

Department of Food Science

University of Guelph

50 Stone Road East

Guelph, Ontario, N1G 2W1

Phone: (519) 824-4120 x53732

E-mail: paul.spagnuolo@uoguelph.ca

**1. Supplementary Methods**

***Materials***

Avocadyne and avocadene (90% purity) were purchased from Microsource Discovery Systems Inc. (CT, USA). Polysorbate (tween) 20, 60 and 80, span 65 and 80, Cremophor EL (Kolliphor ® EL), polyethylene glycol, polyethylene glycol monolaurate, soybean oil, phosphate buffered saline (PBS), 1-heptadecanol, heptadecanoic acid, palmitic acid, oleic acid, penicillin/streptomycin, trypsin solution, p-anisaldehyde stain and formic acid (FA) were purchased from Sigma-Aldrich. Neobee M-5 was a provided as gift from Stepan Company (Northfield, IL). Capryol 90, Larafil M 1944 CS, Labrasol, Transcutol, HP, and Maisine CC were received as a gift from Gattefosse (St-Priest, France). 16-heptadecynoic acid was purchased from Toronto Research Chemicals. Neusilin ULF2 was purchased from Fuji Chemical Industries Inc (NJ, USA). 3-(4,5-dimethylthiazol-2-yl)-5-(3-carboxymethoxyphenyl)-2-(4-sulfophenyl)-2H-tetrazolium inner salt (MTS) reagent was purchased from Promega (Canada). Naproxen, and curcumin were purchased from Caymen chemicals (MI, USA).

***Avocado polyol extraction, purification and analytical characterization***

Avocatin B was extracted from Hass avocado seeds, as originally described by Kashman and colleagues ^1^ with some modifications. Briefly, seeds were air dried, crushed and placed in glass bottles with ethyl acetate in a 2:1 solvent to seed ratio. The bottles were sealed and allowed to rotate on a bench top roller (120 VAC Wheaton Mini Bench Top Roller) for 24 hours and extracts were gravity filtered and the solvent was evaporated using a rotary evaporator. This process was repeated twice and all extracts were combined. The crude extract was purified using flash silica gel chromatography using ethyl acetate as the mobile phase ^2^. Column fractions were analyzed for purity using thin layer chromatography (eluent: ethyl acetate, R_f_ = 0.30) and visualized using p-anisaldehyde stain. Additionally, different ratios of avocadene and avocadyne were blended using commercially available avocadene and avocadyne powders in a 3:2 or 3:1 avocadene:avocadyne ratio in glass scintillation vials.

All purified samples were characterized by ^1^H NMR. Samples were dissolved to 2 mg/mL in deuterated chloroform (CDCl_3_) and transferred to an NMR tube. Samples were analyzed using an Avance 400 MHz spectrometer (Bruker, Canada) and recorded as parts per million (ppm) using CDCl_3_ as internal standard (CDCl_3_: δ 7.24 ppm (^1^H at 400 MHz)). Supplementary Figure 1 shows chemical shifts (δ (ppm)) and coupling constant (*J* (hertz)) for avocadyne, avocadene, avocatin B, 3:2 avocadene—avocadyne, and 3:1 avocadene—avocadyne. Avocadyne: ^1^H NMR (400 MHz, CDCl_3_) δ: 3.93-3.88 (m, 2H), 3.64-3.60 (m, 1H), 3.48-3.44 (m, 2H), 2.49 (br. s, 1H), 2.15 (td, *J* = 7.1 and 2.6 Hz, 2H), 2.06-1.98 (m, 1H), 1.91 (t, *J* = 2.6 Hz), 1.57-1.44 (m, 7H), 1.37-1.24 (m, 20H). Avocadene: ^1^H NMR (400 MHz, CDCl_3_) δ: 5.83-5.73 (m, 1H), 4.98-4.87 (m, 2H), 3.96-3.85 (m, 2H), 3.61 (dd, *J* = 11 and 3.5 Hz, 1H), 3.45 (dd, *J* = 11 and 6.4 Hz), 1.99-1.97 (m, 2H), 1.56-1.14 (m, 43H).

Purity was also validated using ultra-high performance liquid chromatography-mass spectrometry (UHPLC-MS) ^3^. Commercially available avocatin B (Microsource Discovery Systems Inc.) was used to generate standard curves for avocadene and avocadyne and then used to quantify and determine avocadene and avocadyne ratios in the extracted and purified seed samples. All test samples were dissolved in 1:1 methanol-acetonitrile at a concentration of 5.71 mg/mL and then diluted to 0.006 mg/mL using the initial composition of the LC mobile phase (60% water-40% acetonitrile + 0.1% FA). All samples were prepared in duplicate and 10 µL was injected into the UHPLC-MS. Supplementary Figure 2 shows representative LC-MS chromatographs.

***X-Ray powder diffraction***

The crystal structure and polymorphic forms of avocado polyols were assayed by X-ray powder diffraction (Multiflex Powder XRD spectrometer, Rigaku, Tokyo, Japan). The copper X-ray tube (wavelength of 1.54 Å) was run at 40 kV and 44 mA. The measurement scan rate was set at 0.5°/min in the range 2θ = 1−30° at 22−23°C. Peak positions (*d*-spacings) calculated from Bragg’s law were determined by MDI Jade 9 (MDI, Livermore, CA, USA) software. Domain sizes (thickness of the nano-crystal) in angstroms (Å) were obtained using Williamson Hull equation ^4–6^ as outlined below:

$FWHM\left( S \right) x\cos\left( \theta\right)=\frac{k x \lambda}{Size}+(4 x Strain x sin(\theta))$

where k is the Scherrer’s constant (0.94) which depends on the shape of the crystal, λ is wavelength of the x-ray radiation (0.15406 nm), FWHM is the full width of half-maximum intensity expressed in radians, and θ is the diffraction (Bragg) angle in radians. Williamson Hull plots of FWHMcos(θ) versus sin(θ) (for 2θ = 1-30°) were plotted for all samples. The slope was used to determine crystal strain and the y-intercept was used to calculate domain size.

***Cell culture and in vitro cytotoxicity***

Cells were cultured at 37°C with 5% CO_2_. OCI-AML2 cells were cultured in Iscove's Modified Dulbecco's Medium (IMDM; Life Technologies) supplemented with 10% fetal bovine serum (FBS; Sigma) and 1% penicillin/streptomycin (100 U/mL of streptomycin and 100 mg/mL of penicillin; Sigma). TEX cells were similarly cultured except with 15% FBS, 2 mmol/L (mM) L-glutamine (Sigma), 20 ng/mL stem cell factor, and 2 ng/mL interleukin 3 (IL3; Peprotech). AML-2 and TEX cells between passages 5-25 were used for all experiments.

Non-AML cell lines included INS-1 (832/13) rat pancreatic β-cell line, C2C12 mouse skeletal myoblast cell line, Caco-2 human epithelial colorectal adenocarcinoma cells, and HepG2 human hepatocellular carcinoma cells. INS-1 (832/13) cells were cultured in RPMI 1640 medium containing 11.1 mM glucose, supplemented with 10% FBS, 1% penicillin/streptomycin, 2 mM L-glutamine, 1 mM sodium pyruvate, and 50 μM β-mercaptoethanol. INS-1 (832/13) cells between passages 75-100 were used for all experiments. C2C12 myoblast cells were cultured in growth media consisting of low-glucose (5.5 mM) Dulbecco’s Modified Eagles Medium (DMEM; Hyclone, ThermoFisher) supplemented with 10% FBS and 1% penicillin/streptomycin. Differentiation of C2C12 myoblasts into myotubes was induced by switching 90% confluent cells to differentiation media consisting of low-glucose DMEM supplemented with 2% horse-serum and 1% penicillin/streptomycin. Differentiation media was changed every 24 hr for up to 5 days prior to all experimental treatments. C2C12 cells between passages 5-20 were used for all experiments. Caco-2 and HepG2 cells were cultured in DMEM medium containing 25 mM glucose, 10% FBS and 1% penicillin/streptomycin. Caco-2 and HepG2 cells between passages 10-40 were used for all experiments. All cell lines were maintained at 37 °C with 5% CO2 and 95% humidity.

The cytotoxicity of avocado polyols was tested in AML cell lines OCI-AML-2 and TEX. Avocado polyols were delivered in either dimethyl sulfoxide (DMSO) or as SEDDs. For DMSO delivery, all avocado polyols were dissolved in DMSO at a concentration of 11.4 mg/mL, which was then diluted to 0.6 mg/mL in cell culture media as a working stock to treat cells at varying concentrations from 1-29 µg/mL. DMSO vehicle control cells were treated with no more than 0.03% DMSO. For SEDDS delivery, a 2% (w/w) (or 20 mg/mL) SEDDS stock of avocadyne, avocadene, avocatin B, and 3:1 avocadene—avocadyne was diluted to 0.6 mg/mL in cell culture media and cells were treated with the same concentrations as mentioned above. For 3:2 avocadene—avocadyne SEDDS delivery, a 1.5% (w/w) (or 15 mg/mL) stock was used due to the higher stability of these emulsion. SEDDS vehicle control cells were treated with no more than 0.3% control SEDDS (1:1 NeoBee®M5—Tween 80 diluted 10 folds in PBS)

For OCI-AML-2 and TEX cells, 1.25x10^5^ cells/ml were seeded in 96-well plates and treated with test compounds for 72 hours. Cells were then incubated with 20 µL of 3-(4, 5-dimethylthiazol-2-yl)-5-(3-carboxymethoxyphenyl)-2-(4-sulfophenyl)-2H-tetrazolium salt (MTS; Promega; Madison, WI) for two hours at 37 °C and 5% CO_2_. Metabolically active cells express extracellular enzymes which can cleave MTS into a coloured formazan product. The formazan product was then quantified by measuring absorbance at 490 nm using a Biotek Synergy HT spectrophotometer (Biotek; Winooski, VT). For non-AML cell lines INS-1 (832/13), Caco-2, and HepG2, 1x10^5^ cells/ml were seeded in 96 well plates, allowed to attach for 24 hr after which they were treated with test compounds for 24 hr before addition of MTS reagent. C2C12 myoblast cells were seeded in 12-well plates and day 5 differentiated myotubes were treated for 24 hr before addition of MTS reagent. Results were analyzed in GraphPad 6.0 prism software and represent logarithmic transformation of avocado polyol concentrations (µg/mL) and cell viability (% MTS reduction relative to vehicle control) that was fit to a nonlinear regression curve (log(agonist) vs. response-variable slope (four parameters)) to determine inhibitory concentration 50 (IC50). All data represents mean ± SEM from three independent experiments performed in triplicate.

***In vivo pilot pharmacokinetic study***

Ten-week-old, female, C57BL/6J mice were purchased (Jackson Laboratory, Bar Harbor, ME) and allowed to acclimatize for 1 week. After acclimatization, mice were randomly assigned (n=3 per treatment group) to receive an oral bolus dose of 100 mg/kg body weight (b.w.) avocatin B (formulated as 2% w/w SEDDS) or vehicle control (control SEDDS). The gavage volume was 5 mL/kg b.w. After 2 hr and 6 hr post gavage, up to 100 µL of whole blood was drawn per animal via tail bleed and collected in K2EDTA coated tubes (Sarstedt, Canada). At endpoint (24 hr post gavage), animals were euthanized via CO_2_ followed by exsanguination from which 500-800 µL of whole blood was collected and stored as described above. Tissue (inguinal fat pad, gonadal fat pad, liver, pancreas, heart, femur (to obtain bone marrow), and brain) was harvested and flash frozen in liquid N_2_. All blood and tissue samples were then maintained at -80 °C until extraction was performed for the analytical determination of avocadene and avocadyne in whole blood and tissues. The bioanalytical, quantitative LC-MS method and its validation is detailed in Supplementary Information section 6. Results for avocadene or avocadyne quantitation are presented as mean ± S.D., in ng/ml for whole blood or in ng/g wet tissue for tissues.

Pharmacokinetic parameters were calculated using non-compartmental analysis with the PK Functions add-in for Microsoft® Excel (Joel I. Usansky, PhD, Atul Desai, MS and Diane Tang-Liu, PhD, Department of Pharmacokinetics and Drug Metabolism, Allergan, Irvine, CA 92606, USA). The total area under the curve (AUC0−t) was determined with the linear trapezoidal rule from the time of dosing while AUC0−inf was extrapolated to infinity. The elimination rate constant (kel) and plasma concentration half life (t½) were determined by regression of the two terminal data points on the semi-logarithmic concentration versus time plot. The maximal concentration (Cmax) and time at maximal (Tmax) were obtained directly from the concentration versus time plot.

All animal studies were carried in accordance to the regulations of the Canadian Council on Animal Care (CCAC) and with the approval of the Animal Care Committee at the University of Guelph.

***Preparation of solid-SEDDS***

The chosen liquid SEDDS were converted to a solid form (solid-SEDDS) using the solid carrier Neusilin (an amorphous form of magnesium aluminometasilicate which has adsorption capacity and flow enhancing properties ^7^). AVO was heated in the oil/surfactant phase at 200 mg/mL which was then adsorbed to 200 mg Neusilin carrier by mixing and sonication for 30 min. The powder was then reconstituted in 4 ml double-distilled water, centrifuged (1500 x g) for 30 min, and mean droplet size of the supernatant was measured using dynamic light scattering as described above.

**2. Avocado polyol analytical characterization**

**Supplementary Figure 1.** ^1^H NMR spectra of avocado seed polyols. **(A)** avocadyne. **(B)** avocadene. **(C)** avocatin B. **(D)** 3:1 avocadene—avocadyne. **(E)** 3:2 avocadene—avocadyne.

**Supplementary Figure 2.** LC-MS chromatographs of **(A)** avocatin B, **(B)** 3:1 avocadene—avocadyne, **(C)** 3:2 avocadene—avocadyne. Avocadyne and avocadene [M+H]^+^ and [M+H-H_2_O]^+^ mass fragments illustrated by employing an extraction window of 10 mDa.

**3. Powder X-ray diffraction**

**Supplementary Figure 3.** Small and wide-angle powder X-ray diffraction spectra for **(A)** avocadyne, (B) avocadene, **(C)** avocatin B, **(D)** 3:1 avocadene—avocadyne, **(E)** 3:2 avocadene—avocadyne.

**Supplementary Figure 4.** Small and wide-angle powder X-ray diffraction spectra and corresponding domain sizes as obtained from Williamson Hull plots for **(A)** 1-heptadecanol, **(B)** heptadecanoic acid, **(C)** 16-heptadecynoic acid.

of avocadene and avocadyne composition.

**4. SEDDS development and characterization**

| **Oil (% v/v)**  **Supplementary Table 1.** Formulations tested for SEDDS properties | **Surfactant (% v/v)** | **Co-surfactant (% v/v or w/w if specified)** | **Self-Emulsifying Formulation after 1:10 dilution in PBS (Y/N)** | **Z-average (d.nm)** | **Polydispersity Index (PDI)** |
| --- | --- | --- | --- | --- | --- |
| **LONG CHAIN TRIGLYCERIDE OILS** | | | | | |
| Olive oil 50% | Tween 80 50% |  | N |  |  |
| Vegetable oil 50% | Tween 80 50% |  | N |  |  |
| Soybean oil 50% | Tween 80 50% |  | N |  |  |
| Soybean oil 50% | Tween 20 50% |  | N |  |  |
| Castor oil 50% | Tween80 50% |  | N |  |  |
| Avocado oil 50% | Tween 80 50% |  | N |  |  |
| **MEDIUM CHAIN TRIGLYCERIDE OILS** | | | | | |
| NeoBee M5 50% | Tween 80 50% |  | Y | 189 | 0.241 |
| NeoBee M5 50% | CrEL 50% |  | Y | 82 | 0.345 |
| NeoBee M5 50% | Labrasol 50% |  | N |  |  |
| NeoBee M5 50% | Tween 20 50% |  | N |  |  |
| NeoBee M5 50% | Span 80 50% |  | N |  |  |
| NeoBee M5 53% | Tween 80 35% | Maisine 10% | Y | 119 | 0.240 |
| NeoBee M5 45% | Tween 80 45% | Maisine 10% | Y | 100 | 0.370 |
| NeoBee M5 42% | Tween 80 42% | Maisine 16% | Y | 103 | 0.300 |
| NeoBee M5 38% | Tween 80 38% | Maisine 14% | Y | 100 | 0.294 |
| NeoBee M5 47% | Tween 80 47% | Span80 6% | Y | 157 | 0.263 |
| NeoBee M5 45% | Tween 80 45% | Span80 10% | Y | 112 | 0.350 |
| NeoBee M5 43% | Tween 80 43% | Span80 14% | Y | 98 | 0.350 |
| NeoBee M5 42% | Tween 80 42% | Span80 16% | Y | 87 | 0.320 |
| NeoBee M5 40% | Tween 80 40% | Span80 20% | Y | 180 | 0.197 |
| NeoBee M5 38% | Tween 80 38% | Span80 24% | Y | 280 | 0.300 |
| NeoBee M5 50% | Tween 80 15% | Labrasol 35% | Y | 260 | 0.155 |
| NeoBee M5 50% | Tween 80 25% | Labrasol 25% | Y | 253 | 0.190 |
| NeoBee M5 50% | Tween 80 35% | Labrasol 15% | Y | 280 | 0.280 |
| NeoBee M5 50% | Tween 80 35% | Span 65 19% (w/w) | Y | 173 | 0.260 |
| NeoBee M5 50% | Tween 80 35% | Span 65 42% (w/w) | Y | 310 | 0.540 |
| NeoBee M5 45% | Tween 80 45% | Transcutol 10% | Y | 238.8 | 0.238 |
| Coconut oil 50% | Tween 80 50% |  | Y | 195 | 0.219 |
| Coconut oil 50% | CrEL 50% |  | Y | 92 | 0.254 |
| Coconut oil 60% | Labrasol 40% |  | N |  |  |
| Coconut oil 50% | Span 80 50% |  | N |  |  |
| Coconut oil 45% | Tween 80 45% | Transcutol 10% | Y | 233 | 0.230 |
| Coconut oil 45% | Tween 80 45% | Maisine 10% | Y | 193 | 0.225 |
| Coconut oil 42% | Tween 80 42% | Maisine 16% | Y | 100 | 0.294 |
| Coconut oil 38% | Tween 80 38% | Maisine 24% | Y | 333 | 0.363 |
| Coconut oil 47% | Tween 80 47% | Span80 6% | Y | 130 | 0.270 |
| Coconut oil 45% | Tween 80 45% | Span80 10% | Y | 109 | 0.280 |
| Coconut oil 43% | Tween 80 43% | Span80 14% | Y | 76 | 0.350 |
| Coconut oil 41% | Tween 80 41% | Span80 18% | Y | 75 | 0.360 |
| Coconut oil 40% | Tween 80 40% | Span80 20% | Y | 198 | 0.225 |
| Coconut oil 38% | Tween 80 38% | Span80 24% | Y | 165 | 0.291 |
| Coconut oil 50% | Tween 80 15% | Labrasol 35% | Y | 268 | 0.215 |
| Coconut oil 50% | Tween 80 25% | Labrasol 25% | Y | 278 | 0.218 |
| Coconut oil 50% | Tween 80 35% | Labrasol 15% | Y | 310 | 0.280 |
| Labrafil 20% | Labrasol 80% |  | N |  |  |
| Labrafil 47% | Labrasol 47% | Capryol 90 6% | N |  |  |
| Labrafil 47% | Labrasol 47% | Transcutol 6% | N |  |  |
| Labrafil 15% | Labrasol 80% | Capryol 5% | N |  |  |
| Labrafil 10% | Labrasol 80% | Transcutol 10% | N |  |  |
| Labrafil 5% | Labrasol 65% | Transcutol 30% | N |  |  |
| Capryol 90 50% | Tween 80 50% |  | N |  |  |
| Capryol 90 10% | Tween 20 45% | Transcutol HP 45% | Y | 15.11 | 0.12 |
| Capryo l90 10% | Tween 20 30% | Transcutol HP 60% | Y | 105 | 0.538 |
| Capryol 90 20% | Tween 20 40% | Transcutol HP 40% | Y | 186 | 0.528 |
| Capryol 90 10% | Tween 20 60% | Transcutol HP 30% | Y | 12 | 0.128 |
| Capryol 90 47% | Tween 80 47% | Transcutol HP 6% | N |  |  |

**Supplementary Figure 5.** Droplet size and PDI for varying amounts of NeoBee®M5 MCT oil tested for self-emulsifying properties when combined with varying amounts of Tween 80 or Cremophor EL. All SEDDS presented here were diluted 10 fold in PBS. Data represents mean ± SEM of two independent experiments.

**Supplementary Figure 6.** CrEL based SEDDS exert more toxicity than Tween 80 in AML and non-AML cell lines. *In vitro* cytotoxicity of 1:1 NeoBee®M5—Tween 80 or NeoBee®M5—CrEL blank SEDDS in **(A)** AML cell line OCI-AML-2 and non-AML cell lines **(B)** INS-1 (832/13), **(C)** Caco-2, and **(D)** HepG2. Cell lines were incubated with varying concentrations of blank SEDDS. For OCI-AML-2 cells cell viability was measured by the MTS assay after 72 h, whereas for non-AML cells viability was measured after 24 h treatments. All data represents mean ± SEM from two independent experiments performed in triplicate.

**Supplementary Figure 7.** AVO reduces droplet size of coconut oil—Tween 80 and NeoBee M5—CrEL SEDDS. **(A)** Effect of AVO concentration on Z-average of coconut oil—Tween 80 SEDDS over time. Inset: visual appearance of control (blank SEDDs) and AVO containing SEDDS on day 0. **(B)** Polydispersity index of SEDDS described in (A). **(C)** Effect of AVO concentration on Z-average of NeoBee^®^M5—CrEL SEDDS over time. Inset: visual appearance of control SEDDS and AVO containing SEDDs on day 0. **(D)** Polydispersity index of SEDDs described in (C). For A-D, values are means ± SEM of three independent experiments; *p<0.05, **p<0.01, ***p<0.001, ****p<0.0001 compared to control, two-way ANOVA, Dunnett’s *post hoc* test.

**Supplementary Figure 8.** Avocadyne exhibits highest melting point, enthalpy of fusion and melting entropy when incorporated in 1:1 NeoBee®M5—Tween 80 oil phase. Avocado polyols were mixed in 1:1 NeoBee®M5—Tween 80 oil phase at a concentration of 200 mg/mL and analyzed via DSC. **(A)** DSC melting temperatures (left Y-axis) and melting enthalpies (right Y-axis) as a function of avocadene and avocadyne composition. **(B)** Melting entropy, ΔS_m_ as a function of avocadene and avocadyne composition, calculated using experimental enthalpies of fusion and melting temperatures. Values for (A-B) are means ± SD from two independent experiments.

**Supplementary Table 2.** Experimentally determined Ostwald ripening rate and coalescence rate for blank SEDDs (control) and avocado polyol SEDDS (between 1-2% (w/w)) at room temperature

**5. Avocado polyol SEDDS stability**

| SEDDS Description | Ostwald ripening  rate  (nm^3^ h^-1^) | Correlation (r^2^) | Coalescence rate (nm^-2^ h^-1^) | Correlation (r^2^) |
| --- | --- | --- | --- | --- |
| Control | 227.5 | 0.5235 | 1.97 x 10^-8^ | 0.4977 |
| AVO 1% w/w | 73.78 | 0.9526 | 2.31 x 10^7^ | 0.8894 |
| AVO 1.5% w/w | 14.93 | 0.9652 | 6.27 x 10^6^ | 0.9151 |
| AVO 2% w/w | 74.56 | 0.9327 | 1.07 x 10^5^ | 0.8195 |
| Avocadene 1% w/w | 80.61 | 0.9253 | 3.15 x 10^7^ | 0.8251 |
| Avocadene 1.5% w/w | 39.14 | 0.8544 | 7.89 x 10^6^ | 0.9583 |
| 3:1 Avocadene-Avocadyne 1% w/w | 46.58 | 0.9539 | 2.57 x 10^7^ | 0.9156 |
| 3:1 Avocadene-Avocadyne 1.5% w/w | 67.16 | 0.934 | 2.43 x 10^6^ | 0.9532 |
| 3:2 Avocadene-Avocadyne 1% w/w | 31.59 | 0.8808 | 6.72 x 10^8^ | 0.8654 |
| 3:2 Avocadene-Avocadyne 1.5% w/w | 86.94 | 0.9867 | 5.43 x 10^7^ | 0.97 |

**Supplementary Figure. 9.** Ostwald ripening and coalescence contribute to instability of avocado polyol containing SEDDS. Mean particle size distribution (PSD) over four weeks at ambient temperature storage for **(A)** 1% (w/w) AVO emulsion, **(B)** 1.5% (w/w) AVO emulsion, and **(C)** 2% (w/w) AVO emulsion. **(D)** Evidence of thermodynamic stability for fresh 20 mg/mL avocatin B SEDDS during centrifugation test. **(E)** Evidence of thermodynamic stability for fresh 20 mg/mL avocatin B SEDDS during free-thaw test. **(F)** Normal light photomicrograph of crystals in a six month aged and destabilized 2% (w/w) AVO SEDDS (left) re-emulsifying into fine droplets upon brief application of heat (right). For A-C data represents mean PSD from three independent experiments.

**Supplementary Figure 10.** Mean particle size distribution (PSD) over four weeks at ambient temperature storage for **(A)** 1% (w/w) avocadene SEDDS, **(B)** 1.5% (w/w) avocadene SEDDS, **(C)** 1% (w/w) 3:1 avocadene—avocadyne SEDDS, **(D)** 1.5% (w/w) 3:1 avocadene—avocadyne SEDDS, **(E)** 1% (w/w) 3:2 avocadene—avocadyne SEDDS, and **(F)** 1.5% (w/w) 3:2 avocadene—avocadyne SEDDS. Data represents mean PSD from three independent experiments.

**Supplementary Figure 11.** Optical isotropy of SEDDS assessed by normal and polarized light microscopy **(A)** Normal light photomicrograph of control SEDDS (1:1 NeoBee M5—Tween 80) (left) and when viewed between crossed polarizers (right). **(B)** Normal light photomicrograph of 20 mg/mL avocatin B SEDDS (left) and when viewed between crossed polarizers (right). All images were captured at 20x magnification.

**Liquid Chromatography-Mass Spectrometry**

**6. AVO bioanalytical method**

A previously developed LC-MS method for the quantitation of avocadene and avocadyne in avocado seed and pulp ^3^ was further validated for mouse whole blood and tissue. All chromatography and mass spectrometer parameters utilized were the same as previously described ^3^ except all quantitative analysis was performed in high-resolution selected-ion monitoring (SIM) mode which enabled avocadene and avocadyne fragmentation ions ([M+H-H_2_O]^+^) to be detected at low detection limits compared to the LC-MS method.

**Whole blood and tissue extraction and sample preparation**

For whole blood extraction, 100 µL of whole blood was extracted using a modified Folch protocol ^8^. Briefly, 100 µL of whole blood was macerated and mixed in 3 mL of 2:1 chloroform-methanol (v/v) at room temperature after which 0.5 mL of 0.2 M sodium-phosphate (NaHPO_4_) buffer in ddH_2_O (pH 4.4) was added to induce layer separation. After inversion, samples were centrifuged for 5 min at 1500 rcf. The total lipid containing organic layer was collected and an additional 2 mL chloroform was added to the aqueous layer as a wash step and as an additional round of extraction. The second organic layer was combined with the first and the buffer layer was discarded. Chloroform extracts were dried under a gentle stream of nitrogen and stored at 4 °C until sample preparation was required for LC-MS analysis. Blank whole blood (blood from non-treated or control mice) was also extracted the same way for recovery experiments and the generation of matrix-matched AVO standard curves. Modified Folch protocol was also utilized for tissue extraction where 75 mg of flash frozen and pulverized tissue was macerated in 3 mL of 2:1 chloroform-methanol (v/v) and homogenized 40 times with a tissue grinder. 0.5 mL of NaHPO_4_ was then added to induce layer separation and double extraction of organic layer was completed as described before. Tissue chloroform extracts were dried under a gentle stream of nitrogen and stored at 4 °C until sample preparation was required for LC-MS analysis. Tissue from non-treated or control mice were also extracted the same way for recovery experiments and the generation of matrix-matched AVO standard curves. On analysis day, all dried blood and tissue samples were reconstituted in 250 µL of LC starting gradient (60% water-40% acetonitrile + 0.1% FA) and 10 µL of each was injected into the LC-SIM-MS method.

**Method validation**

*Selectivity and Linearity*

The effect of endogenous matrix constituents interfering with retention times of avocadyne and avocadene was assessed by extracting and analyzing individual batches (in duplicates) of blank whole blood or tissue using the extraction procedure and chromatographic/mass spectroscopic conditions described above. Responses of the analyte at the lower limit of quantitation (LLOQ) concentration were compared with the responses in the blank samples. Selectivity was found to be acceptable as no interference from endogenous matrix constituents was found for whole blood and all tissue samples. Linearity was assessed by analyzing six-point calibration curves of AVO in mouse whole blood or tissue extracted as described above. Standard curves were constructed using least-square linear regression of peak area under curve versus nominal standard concentrations. Linearity was assessed by evaluating the slope, intercept and coefficient of determination (r^2^) of two different calibration curves produced on separate analytical days. Supplementary Table 3 highlights linear range and correlation coefficients (r^2^) for whole blood and tissues.

*Accuracy and Precision*

Intra- and inter-day accuracy and precision of the developed method were determined by assaying three concentrations of quality control (QC) samples (for blood matrix: LLOQ = 2 ng/mL, low = 100 ng/mL, and high QC = 1800 ng/mL; for tissue matrix: see Supplementary Table 3) in duplicates on two different analytical days. Precision was reported as percent coefficient of variation (%CV) of replicates within one sample run (intra-assay) or between sample runs (inter-assay). Intra- and inter-assay accuracy was reported as percent relative error (% RE) or the percent deviation of QC replicates from nominal concentration. The acceptance limit for accuracy and precision, at low and high QC concentration levels, were set to 15% RE and 15% CV, respectively. For LLOQ, accuracy and precision acceptance limits were set to below 20% RE and 20% CV, respectively ^9^. See Supplementary Table 3 for accuracy and precision parameters for whole blood and tissue.

*Extraction Recovery and Matrix Effect*

Recovery of avocadene and avocadyne from mouse whole blood and tissue was determined by comparing the peak areas of extracted QC samples (at LLOQ, low and high concentrations as highlighted in Supplementary Table 3) with the peak areas of post-extraction blood or tissue blanks spiked at corresponding concentrations. The matrix effect of mouse whole blood and tissue extract on the ionization of avocadene and avocadyne was evaluated by comparing the peak areas of post-extraction blank whole blood or tissue samples spiked at concentrations of QC samples with the areas obtained by QC samples prepared in solvent (LC starting gradient). This analysis was performed for biological replicates. See Supplementary Table 3 extraction recovery and matrix effect for whole blood and tissue.

**Supplementary Table 3.** AVO bioanalytical method validation parameters

**7. AVO Solid SEDDS**

**Supplementary Figure 12.** Avocado polyol SEDDS can be easily incorporated into a solid carrier. **(A)** Method of solid-SEDDS preparation and characterization. **(B)** Droplet size distribution for control or blank solid SEDDS and AVO solid SEDDS. Data represents mean droplet size distribution from three independent experiments.

**References**

1. Kashman, Y., Neeman, I. & Lifshitz, A. New Compounds from Avocado Pear. *Tetrahedron* **25**, 4617- (1969).

2. Still, W. C., Kahn, M. & Mitra, A. Rapid chromatographic technique for preparative separations with moderate resolution. *J. Org. Chem.* **43**, 2923–2925 (1978).

3. Ahmed, N., Smith, R. W., Henao, J. J. A., Stark, K. D. & Spagnuolo, P. A. Analytical Method To Detect and Quantify Avocatin B in Hass Avocado Seed and Pulp Matter. *J. Nat. Prod.* **81**, 818–824 (2018).

4. Patterson, A. L. The Scherrer formula for X-ray particle size determination. *Phys. Rev.* **56**, 978 (1939).

5. Louer, D. & Audebrand, N. Profile fitting and driffraction line-broadening analysis. *Adv. X-ray Anal.* **41**, 556–565 (1999).

6. Enzo, S., Fagherazzi, G., Benedetti, A. & Polizzi, S. A profile-fitting procedure for analysis of broadened X-ray diffraction peaks. I. Methodology. *J. Appl. Crystallogr.* **21**, 536–542 (1988).

7. Gumaste, S. G., Freire, B. O. S. & Serajuddin, A. T. M. Development of solid SEDDS, VI: effect of precoating of Neusilin® US2 with PVP on drug release from adsorbed self-emulsifying lipid-based formulations. *Eur. J. Pharm. Sci.* **110**, 124–133 (2017).

8. Folch, J., Lees, M. & Sloane Stanley, G. H. A simple method for the isolation and purification of total lipides from animal tissues. *J Biol Chem* **226**, 497–509 (1957).

9. Zimmer, D. New US FDA draft guidance on bioanalytical method validation versus current FDA and EMA guidelines: chromatographic methods and ISR. *Bioanalysis* **6**, 13–19 (2014).
